# Size and charge effects of metal nanoclusters on antibacterial mechanisms

**DOI:** 10.1186/s12951-023-02208-3

**Published:** 2023-11-15

**Authors:** Hanny Tika Draviana, Istikhori Fitriannisa, Muhamad Khafid, Dyah Ika Krisnawati, Widodo, Chien-Hung Lai, Yu-Jui Fan, Tsung-Rong Kuo

**Affiliations:** 1 Graduate Institute of Nanomedicine and Medical Engineering, College of Biomedical Engineering, Taipei Medical University, Taipei, 11031 Taiwan; 2 International PhD Program in Biomedical Engineering, College of Biomedical Engineering, Taipei Medical University, Taipei, 11031 Taiwan; 3 Department of Nursing, Faculty of Nursing and Midwivery, Universitas Nahdlatul Ulama Surabaya, Surabaya, 60237 East Java Indonesia; 4 Dharma Husada Nursing Academy, Kediri, 64117 East Java Indonesia; 5 Department of Health Analyst, Faculty of Health, Universitas Nahdlatul Ulama Surabaya, Surabaya, 60237 East Java Indonesia; 6 Sekolah Tinggi Teknologi Pomosda, Nganjuk, 64483 East Java Indonesia; 7 Department of Physical Medicine and Rehabilitation, School of Medicine, College of Medicine, Taipei Medical University, Taipei, 11031 Taiwan; 8 Department of Physical Medicine and Rehabilitation, Taipei Medical University Hospital, Taipei, 11031 Taiwan; 9 Taipei Neuroscience Institute, Taipei Medical University, Taipei, 11031 Taiwan; 10 School of Biomedical Engineering, Taipei Medical University, Taipei, 11031 Taiwan; 11 Center for Precision Health and Quantitative Sciences, Taipei Medical University Hospital, Taipei, 11031 Taiwan; 12 Precision Medicine and Translational Cancer Research Center, Taipei Medical University Hospital, Taipei, 11031 Taiwan; 13 Stanford Byers Center for Biodesign, Stanford University, Stanford, CA 94305 USA

**Keywords:** Nanomaterials, Metal nanoclusters, Size, Ligand, Surface charge, Antibacterial mechanism

## Abstract

Nanomaterials, specifically metal nanoclusters (NCs), are gaining attention as a promising class of antibacterial agents. Metal NCs exhibit antibacterial properties due to their ultrasmall size, extensive surface area, and well-controlled surface ligands. The antibacterial mechanisms of metal NCs are influenced by two primary factors: size and surface charge. In this review, we summarize the impacts of size and surface charge of metal NCs on the antibacterial mechanisms, their interactions with bacteria, and the factors that influence their antibacterial effects against both gram-negative and gram-positive bacteria. Additionally, we highlight the mechanisms that occur when NCs are negatively or positively charged, and provide examples of their applications as antibacterial agents. A better understanding of relationships between antibacterial activity and the properties of metal NCs will aid in the design and synthesis of nanomaterials for the development of effective antibacterial agents against bacterial infections. Based on the remarkable achievements in the design of metal NCs, this review also presents conclusions on current challenges and future perspectives of metal NCs for both fundamental investigations and practical antibacterial applications.

**Graphical Abstract:**

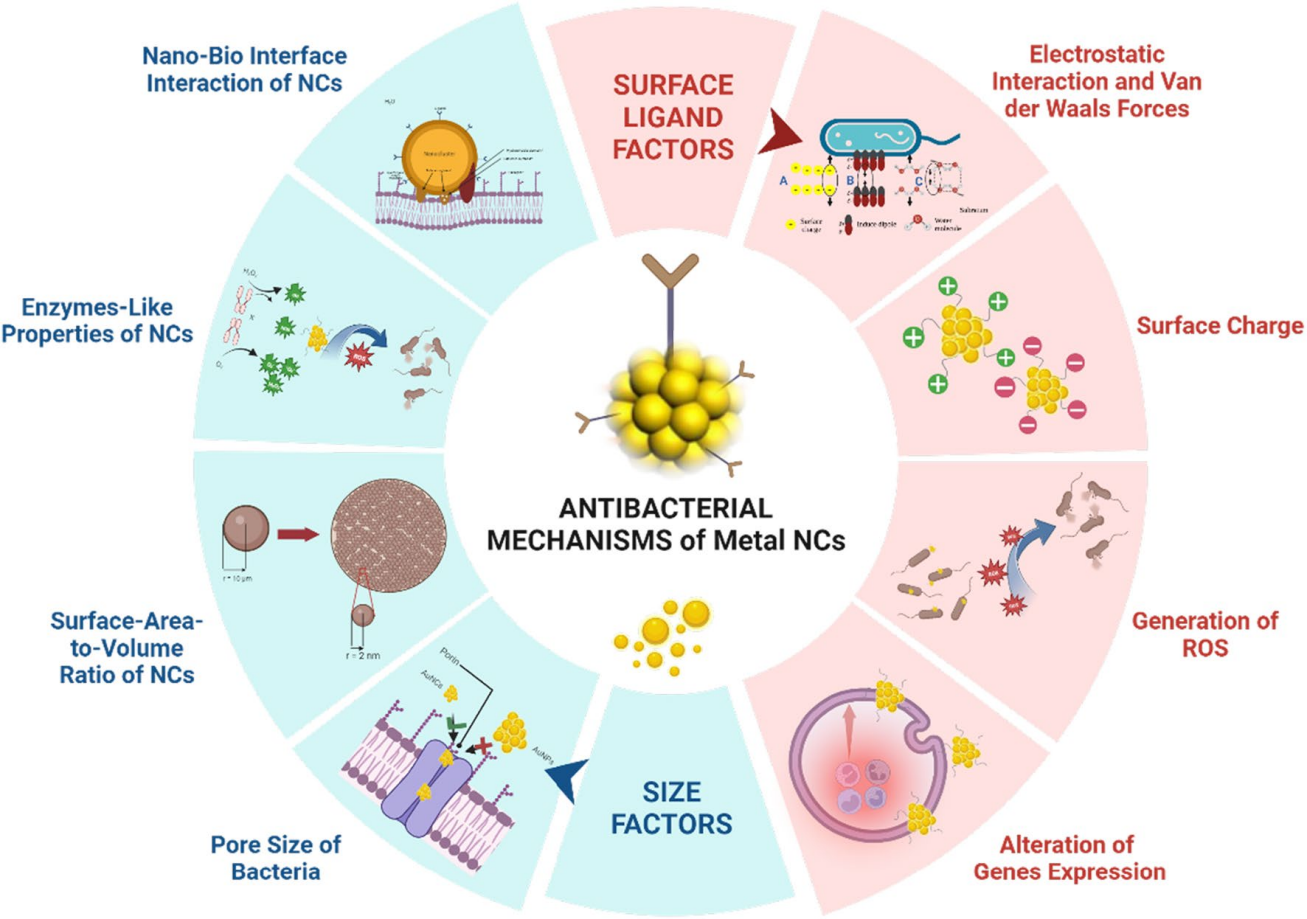

## Introduction

Bacterial infections are a severe concern for human health, and have been further aggravated by rising antibiotic resistance [1–4]. These infections can cause significant morbidity and mortality, thus emphasizing the importance of quickly and efficiently identifying and treating pathogenic bacteria [5]. In general, antibacterial resistance mechanisms are classified as follows: (1) restriction of drug absorption, (2) drug inactivation, (3) drug target modification, and (4) active drug reflux [6–10]. The mechanism of antibiotic resistance is based on the structure of the bacterium itself. Bacteria are classified into two types based on their structure: gram-negative and gram-positive bacteria. Gram-negative bacteria have three major components: the outer membrane (OM) which contains lipopolysaccharides (LPSs), the peptidoglycan (PG) cell wall, and the inner membrane (IM). Meanwhile, gram-positive bacteria lack an OM but have a thicker PG layer [11]. As a result, to combat bacterial infections, technologies must be developed. Nanotechnology is one method that has been developed to combat the problem of bacterial infections [12–15].

The discovery of antibacterial nanomaterials such as metals, metal oxides, carbon, quantum dots, peptides, and polymer-based nanostructures has provided novel opportunities for combating the bacterial infection crisis [16–22]. Among antibacterial nanomaterials, metal nanoclusters (NCs), which have very small sizes and are made up of several to many metal atoms, are gaining popularity for antibacterial applications [12–15]. Metal NCs exhibit antibacterial activity due to a variety of benefits including an extremely large surface area, small size, morphological control, easy surface modification, and physiochemical features (such as distinctive optical, electromagnetic, and catalytic properties) [23–26]. Gold NCs (AuNCs), silver NCs (AgNCs), copper NCs (CuNCs), and alloy NCs are examples of metal NCs that have been used as antibacterial agents [27–29]. Among all types of metal NCs, AgNCs and CuNCs show more-prominent antibacterial activities than the others because silver and copper elements have broad-spectrum antibacterial properties [30–32]. Unfortunately, the antibacterial efficacies demonstrated by these two types of NCs are not necessarily accompanied by biocompatibility in mammalian cells, raising questions about their safety when applied clinically. Because of their biological inertia and high stability, AuNCs have a competitive advantage over AgNCs and CuNCs. AuNCs were also found to be highly biocompatible in living systems [33, 34].

When investigating metal NCs as antibacterial agents, it is vital to investigate the antibacterial mechanisms and elements that influence them. Based on their specific physiochemical characteristics, metal NCs have three primary bactericidal routes. Initially, membrane disruption is caused by bonds between the nanomaterials and portions of the bacterial surface. Second, nanomaterial interactions with numerous biomolecules in cells disrupt biomolecular processes such as DNA replication, protein synthesis, and molecular catalysis. Eventually, the production of oxidative stress and electrolyte imbalances in cells induce the death of bacterial cells due to the absence of normal cellular functions [35]. The size and shape of the metal NCs impact how well they can penetrate bacterial cells during the antibacterial reaction. When metal NCs attach to a cell membrane, the contact surface area and charge of the metal NCs are two key elements causing membrane damage [36–38]. Differences in the composition and charge of metal NCs also affect the production of oxidative stress, which causes bacterial cell death [39].

In this review, we summarize the two main factors that influence antibacterial mechanisms in bacteria, namely the size and surface charge of metal NCs. To begin with, we discuss how the size of NCs affects antibacterial applications, what factors influence the antibacterial effects based on their size against both gram-negative and gram-positive bacteria, and examples of applications of metal NCs that are affected by the size of the NCs. Following that, we outline the surface charge impacts of NCs in antibacterial applications, the mechanisms that occur when NCs are negatively or positively charged, and applications of both types of NCs as antibacterial agents. Thereafter, a brief overview of the relationship between antibacterial activity and various properties of metal NCs should be further studied so that it can be used in the future to aid in designing and synthesizing nanomaterials to develop antibacterial agents that are effective in dealing with bacterial infections.

## Overview of physicochemical properties of NCs

Metal nanoclusters (NCs) have unique physicochemical properties that make them promising for various applications, including therapeutics, sensing, catalysis, and bioimaging. Some of the physicochemical features of metal NCs are ultrasmall size, rich and tailorable surface chemistry, molecule-like properties, good renal clearance, good photostability, tumor targeting, metallic properties, optical properties, catalytic activity, size-dependent properties, biocompatibility, and self-assembly. Metallic properties like electron delocalization in bulk metals, provide them with mechanical ductility, light reflectivity, and high electrical and thermal conductivity [40, 41]. The optical characteristics of metal nanoclusters are linked to their impressive capacity for absorbing light in the visible region. Owing to pronounced quantum confinement effects, metal nanoclusters exhibit distinctive electronic characteristics, including strong photoluminescence (PL), electron dynamics reminiscent of molecules, discrete optical absorption, and non-linear optical behavior. In summary, the optical characteristics of metal NCs are influenced by several factors, including shape, size, constituent elements, and dielectric environment [42–45]. Furthermore, the catalytic activity of metal nanoclusters which exhibit high catalytic performance can lead to enhanced stability, activity, and selectivity compared to bulk metal catalysts. The catalytic activity of metal NCs refers to their ability to accelerate chemical reactions by providing an active surface for the interaction of reactant molecules [46–48]. The size of metal NCs can also significantly impact their catalytic properties. The unique dependent characteristics of metal NCs have been demonstrated in various catalytic reactions, such as carbon–carbon bond formation, oxidation, and hydrogenation. Studies have shown that the photocatalytic activity of gold nanoclusters increases with decreasing size. Additionally, the size-dependent properties of metal NCs can also influence their optical, magnetic, and photoluminescent properties, which can further enhance their catalytic performance [42, 49]. Metal NCs also show biocompatibility properties that refer to their ability to interact with biological systems in a safe and non-toxic manner. The small size of metal NCs allows for the efficient cellular uptake and can reduce their toxicity compared to larger nanoparticles. Additionally, the surface chemistry of metal NCs can be tailored through ligand engineering, which can further enhance their biocompatibility and enable specific interactions with biological targets [50–52]. Lastly, the self-assembly of metal NCs is one of unique phenomenon that arises from their small size, atomically precise structures, and the interplay of various non-covalent interactions. The self-assembly of metal NCs can lead to the formation of ordered structures or aggregates with distinct properties and functionalities that are different from their individual or randomly aggregated counterparts. This process is driven by factors such as solvent conditions, temperature, and the presence of ligands or biomolecules, which can be tailored to control the structure, shape, and dimensions of the resulting self-assembled architectures [53–55]. In summary, the physicochemical properties of metal NCs are diverse and can be tailored through design and engineering approaches. Metal NCs show great potential as advanced materials due to their unique properties and the ability to tailor their physicochemical properties for various applications.

## Overview of NC sizes in antibacterial applications

**Fig. 1:**
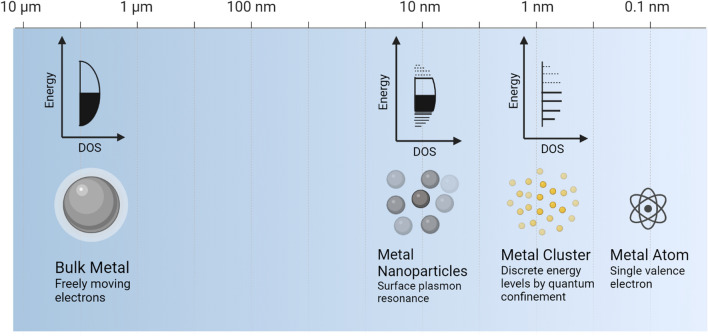
Effects of the size of a metal on it electrical and optical properties

One thing that impacts NCs’ effectiveness as antibacterial materials is their particle size. In the nanoscience field, properties of nanoparticles (NPs) are one of the most intriguing aspects which can be influenced by size changes. These significant size differences have effects not only on the electrical and optical properties of NPs but also on interactions with the environment and biology, such as the transport of NPs via water systems and interactions between NPs and cells [56]. Because of their smaller size, NPs exhibit different chemical and physical properties compared to their bulk substance. Antibacterial activity is typically higher in NPs that are smaller in size [57]. Ultrasmall NPs, also known as NCs, have a core size of < 3 nm and are the only ones among all of the designed NPs developed today that serve as unique platforms for researching size dependencies at an incredibly small scale. The exact control of nanomaterials with sizes down to the atomic level is highly valued since a heterogeneous size distribution can lead to a great deal of uncertainty when it comes to recreating nanomedicines on a large scale with the same level of therapeutic efficacy [58–60]. One study conducted by Zheng et al. reported that AuNCs with different atomic numbers exhibited molecule-like compared to size-dependent antimicrobial behaviors with comparable effectiveness. This indicated the unique molecule-like features of ultrasmall AuNCs [61]. The electrical and optical properties of a metal are influenced by its size, as shown in Fig. 1. When the size of the metal is reduced to a nanoscale or less, which is equivalent to a few or fewer atoms, the band structure becomes fragmented and is separated into several energy levels in a manner that is analogous to the energy levels of molecules. Consequently, it can be seen that metallic NCs have properties similar to molecules, and they no longer display plasmonic capabilities [62].

The size of NCs can affect how they kill bacteria and the bacterial mechanisms. The smaller the size, the greater the ability to penetrate bacterial cell bodies. This is because the ratio of surface area to volume is greater for smaller sizes than for larger sizes, making them more toxic. A large surface area which is one of the size-dependent antibacterial effects, causes NCs to contact with bacterial cells and allows them to reach more cytoplasm than larger NPs. NPs come in a wide variety of shapes and sizes. However, those with the smallest diameters and highest zeta potentials are the ones most likely to cause interference with cell membranes and, as a result, produce a loss of cell viability [63, 64]. As their size is comparable to biological molecules, such as large protein complexes, it is hypothesized that NCs can engage in subcellular reactions. This can enhance the creation of reactive oxygen species (ROS), which can damage and inactivate vital macromolecules such as DNA, proteins, and lipids [65, 66]. There are four important groups of things that affect the antibacterial effect based on the size of the particles. These are explained in more detail below.

### Pore sizes of bacteria

Gram-positive and gram-negative bacteria each have their unique cell structures. Cells of gram-negative bacteria, in particular, are subdivided into several compartments. There are three primary layers, the IM, the PG cell wall, and the OM. The OM is the most superficial layer. Gram-negative bacteria are distinguished from gram-positive bacteria by the presence of an OM; on the other hand, gram-positive bacteria do not have this structure [67]. Antibiotics, disinfectants, cationic peptides, and bacteriocins are a few examples of harmful elements found in the external environment, against which the OM defends, as it acts as a physical and mechanical barrier. Proteins found in the OM mediate the uptake of cells, either passively or actively, of tiny molecules necessary for cell growth and function [68]. The plasma membrane of gram-negative bacteria is encased within the cell’s OM, which shields it from damaging effects of the surrounding environment. Because it contains specialized proteins that can cross membranes and are referred to as “porins”, this membrane serves as a filtering barrier. Gram-negative bacteria are the ones that led to the discovery of porins. Porins are huge water-filled pores that span the OM of gram-negative bacteria [69].

**Fig. 2:**
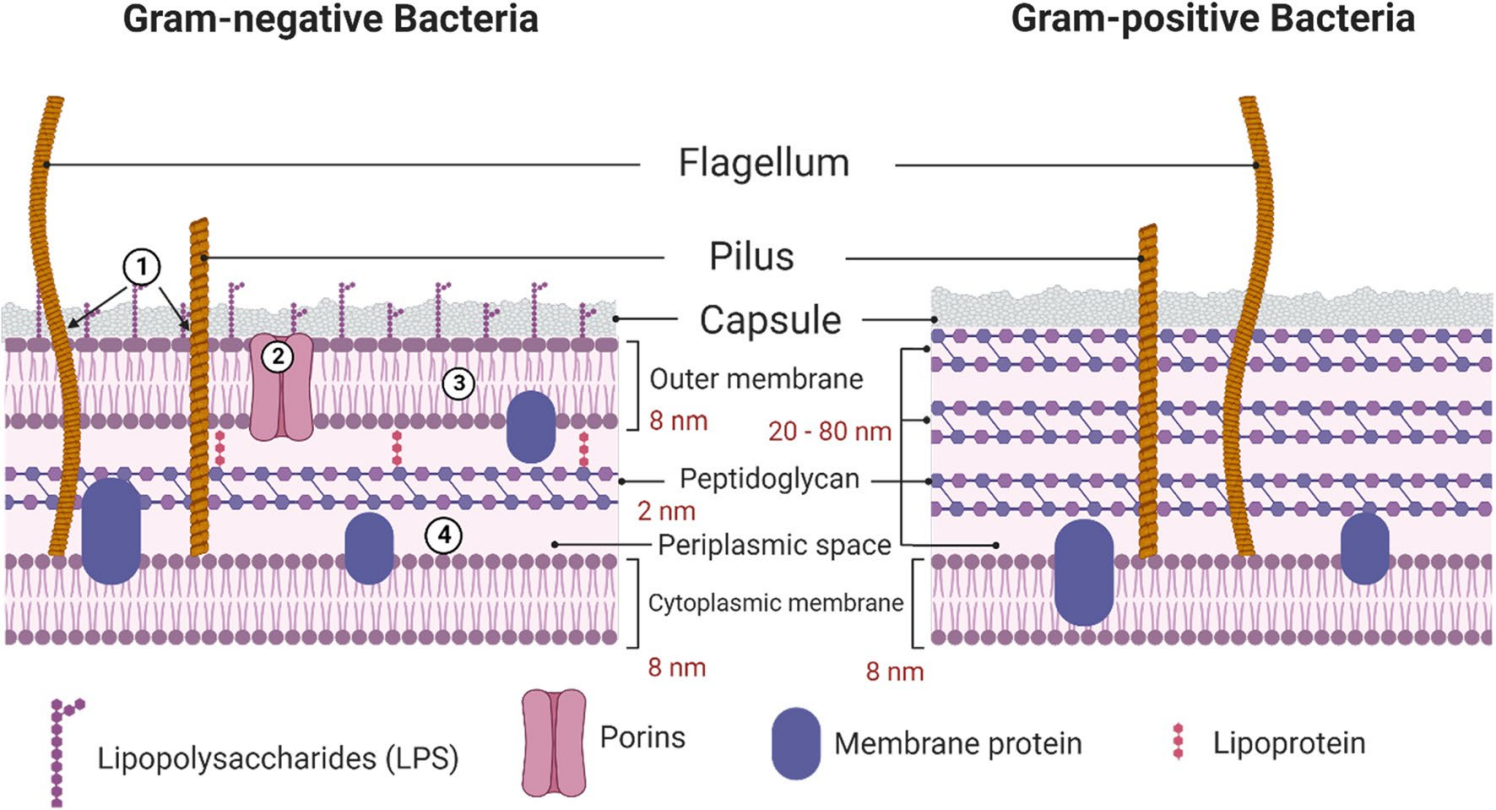
The pathway and the involved physicochemical challenges while crossing the respective barriers

Porins in the OM have no binding affinity toward solutes allowed to pass through. These proteins generally enable the passive diffusion of small hydrophilic solutes with a size exclusion limit of 600 Da [70]. To get to the target in gram-negative bacteria, the first thing that needs to be done is to pass through the OM, which is the layer of negatively charged LPS (1). Porins (2) provide passage for hydrophilic molecules of a smaller size, while the outer hydrophobic layer allows passage of hydrophobic molecules of a greater size. Both types of molecules can eventually reach the periplasmic region (3). To reach their intended destination in the cytoplasm, molecules must traverse a second hydrophobic barrier (4). Hydrophobic molecules have the potential to spread laterally, be captured by an efflux pump, and then be expelled back into the environment. Every step is accomplished through passive diffusion, except for the efflux pumps, which use promotive forces or ATP [71]. The schematic pathway of crossing the membrane of gram-positive and gram-negative bacteria is shown in Fig. 2.

Unlike gram-negative bacteria, gram-positive bacteria are characterized by their thick and continuous cell wall structure known as a sacculus, which primarily consists of peptidoglycan. This multi-layered cell wall, composed of proteins and molecules, serves as a protective barrier against various forms of damage to the bacteria. The peptidoglycan layer within gram-positive bacteria is notably robust, with a thickness ranging from 20 to 80 nm, signifying a substantial increase in thickness compared to the cell wall of gram-negative bacteria, which is typically only about 2 to 3 nm thick [72]. The peptidoglycan is comprised of sequences of alternating MurNAc and GlcNAc components, which are cross-linked via the pentapeptide units. Teichoic acids are unique to Gram-positive bacterial structures and consist of two primary elements. The first one is lipoteichoic acid (LTA), which is a lipid found within the cell membrane. The second element is wall teichoic acid (WTA), which is covalently linked to peptidoglycan. All types of Gram-positive bacteria are surrounded by a single-unit lipid membrane [73]. For a long time, it was believed that Gram-positive bacteria did not possess porin channels. Porin channels are proteins located in the outer membranes of Gram-negative bacteria, creating conduits for the passage of different substances, including antibiotics [74]. However, research has shown that porins are also found in gram-positive bacteria. They exhibit transport functions analogous to those of gram-positive bacteria, although their distribution is more restricted than that in gram-negative bacteria. These formations might have a function in conveying particular molecules or ions, but their precise roles remain incompletely comprehended. Some gram-positive bacteria, such as mycobacteria, have particular lipids attached to porins within their cell walls. Therefore, while it was once believed that gram-positive bacteria lacked porin channels, it is now known that some of them may possess similar structures with different function [69, 75].

**Fig. 3:**
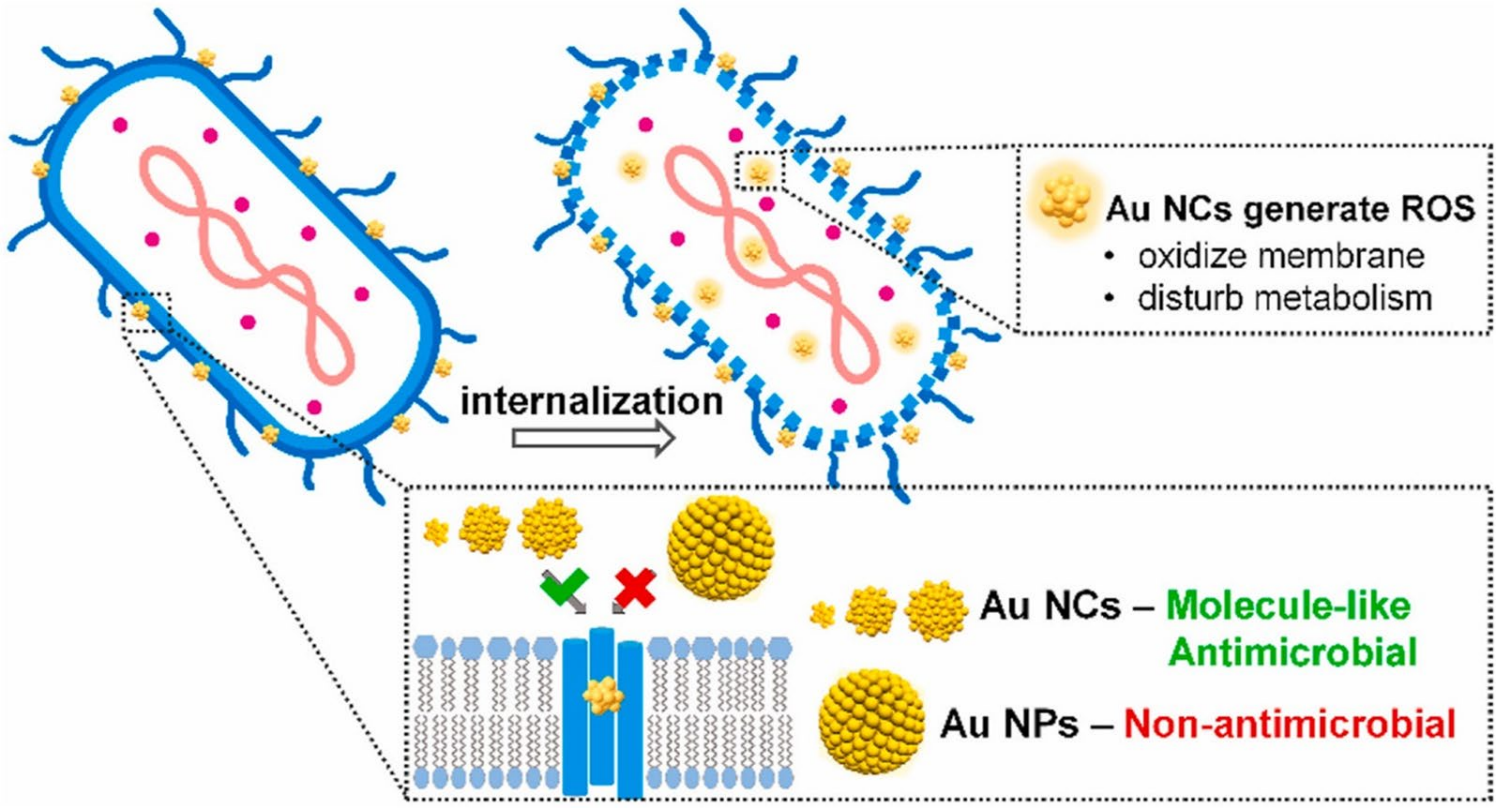
Schematic representation of the antimicrobial mechanism of gold nanocarriers (AuNCs) and gold nanoparticles (AuNPs) with p-mercaptobenzoic acid (MBA) as a ligand. Reproduced with permission from ref [61]. Copyright 2021 Elsevier

Membrane pores are one of the deterministic features that can affect nanomaterial uptake, as nanomaterials can diffuse easily into bacteria. Pores, such as those created by porin trimers, typically have a diameter of 1.2–2 nm, which enables the rapid and easy internalization of ultrasmall AuNCs (< 2 nm). Zheng et al. conducted a study comparing the antibacterial properties of two AuNPs and three AuNCs utilizing p-mercaptobenzoic acid (MBA) as a ligand. According to findings of that investigation, the antibacterial impacts of the three types of AuNCs were higher than when treated with AuNPs. This demonstrated that AuNCs can be easily internalized by bacteria via simple diffusion through small pores in the cell wall. In contrast, the larger sizes of AuNPs make it extremely difficult for them to enter the pores, resulting in low uptake into bacterial cells. The "size cutoff" in the internalization process within bacteria may explain the remarkable difference in antibacterial efficacies between AuNPs and AuNCs (Fig. 3) [61].

Wang et al. also conducted a study employing AuNCs with 6-mercaptohexanoic acid (MHA) as the ligand. In their study, both gram-negative and gram-positive bacteria were subjected to varying concentrations of MHA AuNCs to determine these compounds’ antibacterial properties. The antibacterial effect created by AuNCs treated with a concentration of 150 μM was most effective against gram-negative bacteria. Gram-positive bacteria, on the other hand, showed no signs of being inhibited by the antibacterial properties of AuNCs, even at very high concentrations (150 μM). This may be because of the decreased binding efficacy between AuNCs and gram-positive bacteria. To confirm that the antibacterial effect seen was a direct result of the ultrasmall structure, the researchers tested both AuNCs MHA and AuNPs on *Escherichia coli*. Compared to the use of AuNCs, the antibacterial impact was much lower when treated with AuNPs. This demonstrated that overall, the ultrasmall nature of AuNCs was responsible for the improved antibacterial action compared to other ligands or structures [76].

### The surface-area-to-volume ratio of NCs

**Fig. 4:**
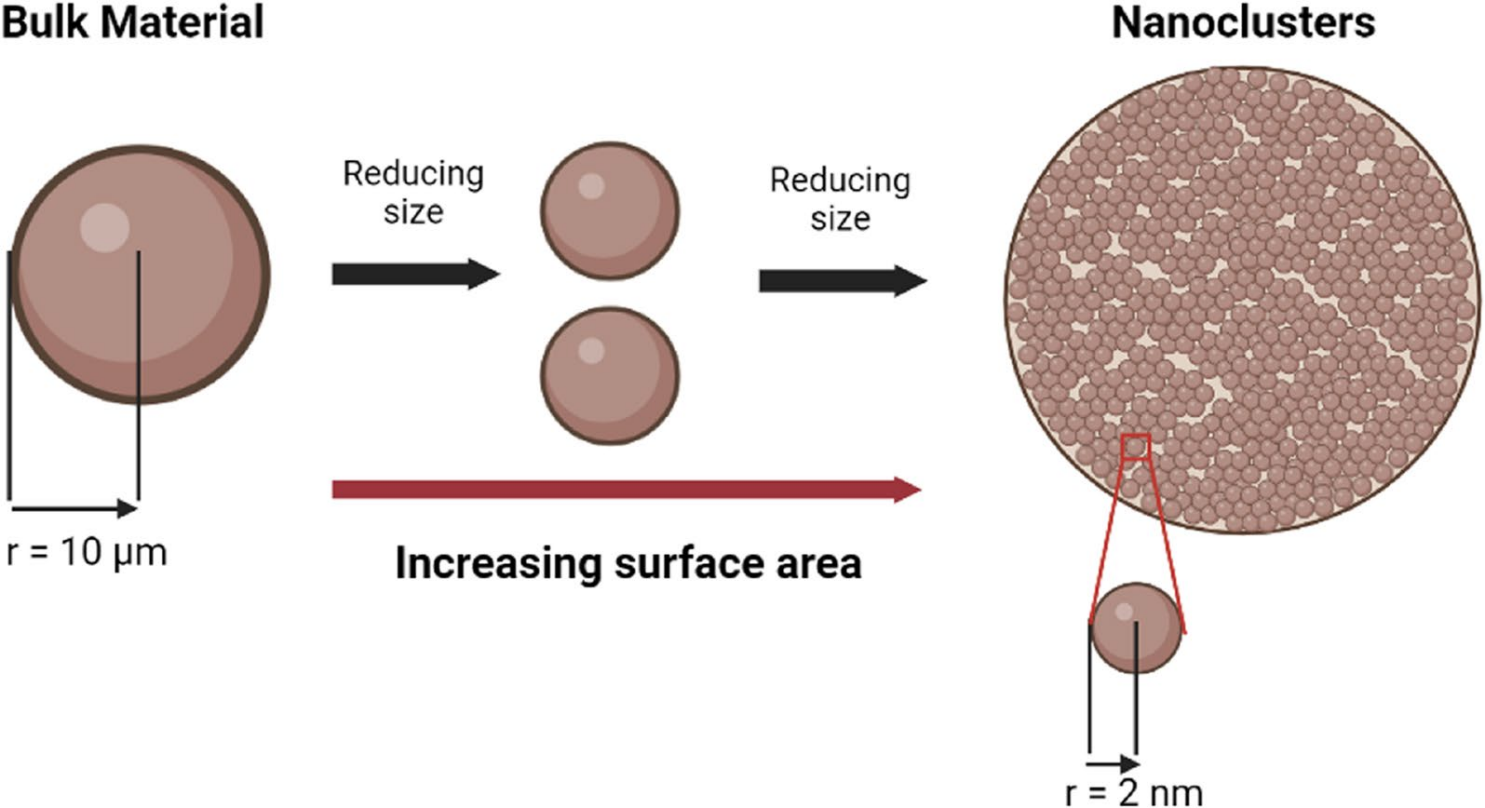
Illustration of the difference surface-area-to-volume ratios between bulk material and nanoclusters

Because NCs have such small particle sizes, the surface-to-volume ratio of a given quantity of particles is improved. This, in turn, boosts the antibacterial properties of NCs and reveals how they interact with and penetrate bacteria. As the size of the particles becomes smaller, the specific surface area of the NCs increases, which allows for a greater amount of contact between the material and the environment [77, 78]. In a given volume, if there is a greater ratio of surface area to volume, the reaction there will be both more rapid and more intense [63]. Figure 4 illustrates how surface area increases as the particle size decreases.

Some research demonstrated that the antibacterial efficacy will improve if the particle size is reduced. At the same time, the ratio of surface area to volume increases. Zheng et al. performed research showing that ultrasmall AuNCs contain strong wide-spectrum antibacterial activity. However, this activity was not present in larger AuNPs counterparts, which were used for comparison. In their research, they used MHA as the ligand, and they treated gram-positive (*Staphylococcus aureus*) and gram-negative (*E. coli*) bacteria with the same concentration (0.1 mM based on Au atoms) of Au_25_MHA_18_ and Au-MHA NPs. They found that the treatment inhibited both types of bacteria. The as-prepared AuNPs only generated a low killing impact on *S. aureus*, which was only 3% of the *S. aureus* population. Nevertheless, the AuNCs efficiently killed approximately 95% of the *S. aureus* population after exposure for 2 h. This was also true for gram-negative bacteria, like *E. coli*, where AuNCs eradicated most bacterial populations. After 2 h of treatment, they found that approximately 96% of the bacterial population had been eliminated. However, treatment with larger-sized AuNPs only successfully eliminated approximately 2% of the bacterial population. The vast disparity in size between AuNCs and NPs gave rise to the hypothesis that the MHA ligand densities on their surfaces were distinct. Compared to the equivalent AuNPs, AuNCs had a greater surface-to-volume ratio, enabling them to have a higher MHA-to-Au ratio on their surface (per particle). Due to the ultrasmall size of the AuNCs, they exhibited substantial antibacterial activity that was effective against a broad spectrum of pathogens. This suggests that AuNCs had a high surface-to-volume ratio [79].

Research was also carried out by Ye et al. to improve the antimicrobial efficacy of nanomedicines. This was accomplished by combining highly luminous silver NPs (AgNPs) with the homogeneous conjugation of an antimicrobial peptide (Dpep-AgNCs). An increase in activity occurred the in size range of 5–100 nm, when it is noted that the size of the AgNPs decreased. Opep, which possesses similar antimicrobial activity, was chosen to synthesize AgNPs to demonstrate the effect of the size-dependent antibacterial performance (Opep-AgNPs). Compared to Dpep-AgNCs, which had an average size of only 1.25 nm, the average size of Opep-AgNPs was approximately 5 nm. According to the findings of that research, the antibacterial activity of Dpep-AgNCs was significantly higher than that of Opep-AgNPs. This may have been because Dpep-AgNCs feature extra-small particles, which substantially boosted the likelihood of penetration into the bacterium. A mice model was used to further illustrate the antibacterial influence of Dpep-AgNCs and their practical applicability. This was done based on the favorable results that had previously been established. Opep-AgNPs were only able to heal 80% of wounds. However, Dpep-AgNCs were able to heal 91% of wounds. This was determined by infecting mice with *E. coli*. After that, it was demonstrated that Dpep-AgNCs accelerated the wound closure of mice, with 91% of wounds healed. This implies that Dpep-AgNCs displayed an immediate healing effect, indicating that they had the potential to be used as an alternative to antibiotics [80].

### Enzyme-like properties of NCs

NCs are a viable possibility for artificial enzymes because enzymes are highly successful at catalyzing various processes with excellent substrate selectivity, activity, and yields, even in moderate conditions [81]. NCs are materials that can efficiently catalyze the conversion of substrates, and their ability to effectively and efficiently catalyze the conversion of substrates is distinctive. They do this by adhering to the same kinetics and mechanisms as natural enzymes when operating in physiological environments [82]. Nanomaterials based on cerium oxide, carbon, metal oxides, metals, and iron oxides are all examples of different nanomaterials that can imitate natural enzymes [83–86]. The size of NCs affects at least one of their enzymatic activities. In the case of gold nanomaterials, the enzyme-like activity of AuNCs will be significantly higher than that of AuNPs. According to the findings of that research and examination, it was determined that changes in the crystal planes of the substances were responsible for differences in the enzyme-like activities of the substances. The performance of a catalytic reaction suffers when the particle size and roughness of the particle surface increase. So, the smaller the particle size, the bigger the surface area-to-volume ratio. As a result, the increased number of metal atoms on the surface leads to a rise in the catalytic activity of the particles [87]. It was reported that NCs act as enzymes, mimicking peroxidase-like or oxidase-like activity that converts hydrogen peroxide (H_2_O_2_) into ROS capable of killing both gram-positive and gram-negative bacteria [88, 89]. In general, the enzyme-like activities of NCs can be divided into four categories including peroxidase-like, oxidase-like, superoxide dismutase (SOD)-like, and catalase-like enzyme activities [90]. However, only peroxidase- and oxidase-like enzymes, which have a stronger impact on antimicrobial activity, are discussed in detail here.

### Peroxidase-like properties of NCs

It is well-known that naturally occurring peroxide systems possess antibacterial abilities. Peroxidase alone has no antimicrobial action. Peroxidases are a category of enzymes capable of catalyzing the oxidation of H_2_O_2_ to hydroxyl radicals (⋅OH). Antimicrobial peroxidase systems require three essential components: a specific peroxidase enzyme, H_2_O_2_, and a substrate that can be oxidized [91]. AuNCs can adsorb H_2_O_2_ onto their surfaces and break down O–O bonds of H_2_O_2_ into dihydroxy radicals. AuNCs can also stabilize the formed hydroxyl radicals through a partial electron exchange interaction [87]. In general, hydroxyl radicals are one of the most damaging ROS. Not only do they break down proteins, nucleic acids, polysaccharides, and other components of the bacterial biofilm, but they also break down the structural integrity of the bacteria, ultimately resulting in bacterial death. In the presence of NCs, only low concentrations of hydrogen peroxide (0.5% or less) are needed to significantly increase the antibacterial efficacy [88].

### Oxidase-like Properties of NCs

**Fig. 5:**
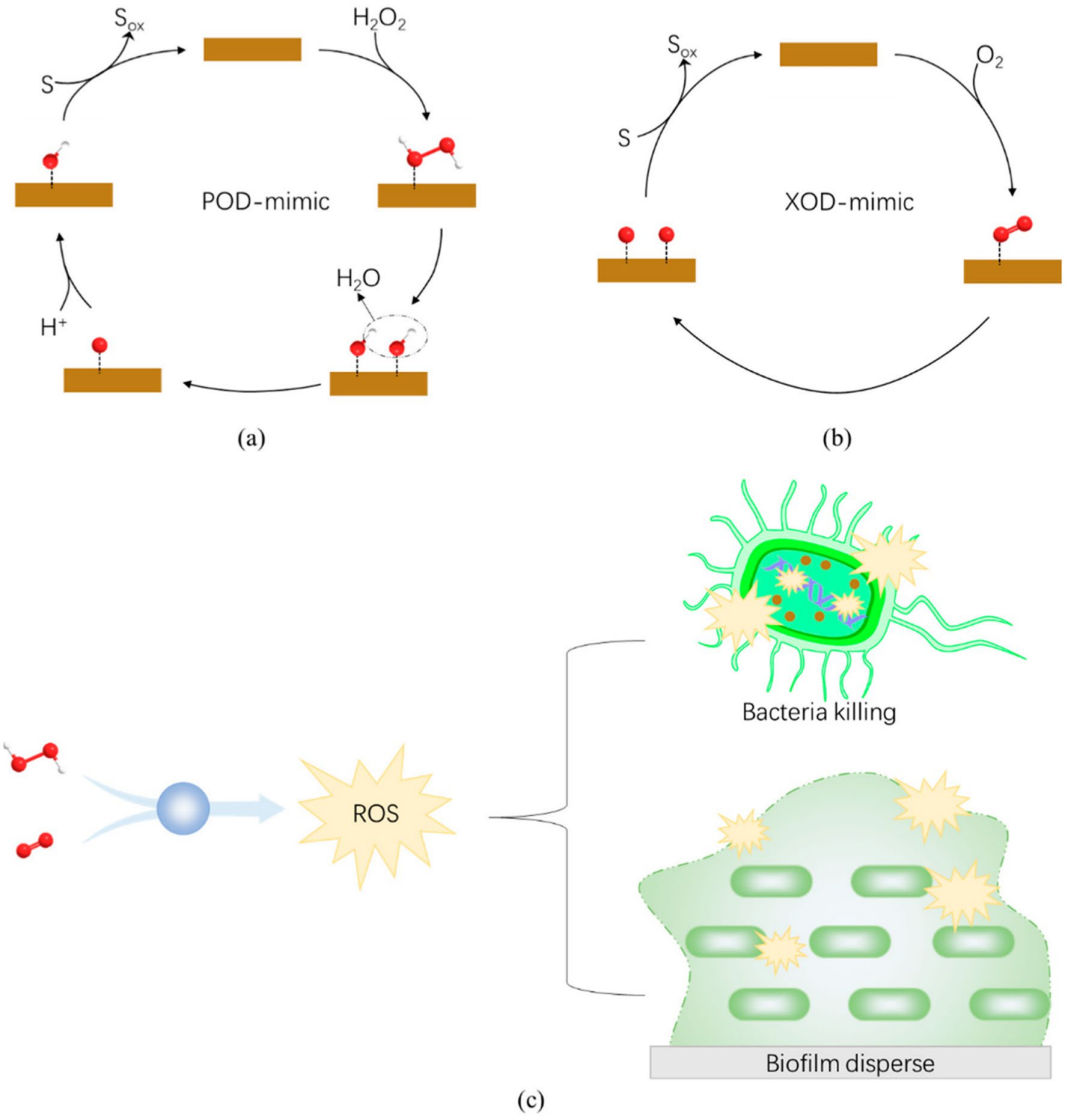
Illustration of catalytic reactions and antibacterial mechanisms of nanoclusters with peroxidase- or oxidase-like enzyme activities. (**a**) Nanozymes exhibiting peroxidase-like activity facilitate the reduction of H_2_O_2_, generating highly reactive hydroxyl radicals (·OH). (**b**) Nanozymes with oxidase-like activity catalyze the conversion of O_2_ to singlet oxygen (^1^O_2_) or even single oxygen atoms. Both ·OH and ^1^O_2_ serve as potent oxidants, leading to the oxidation of the substrate (S) to ox-substrate (Sox), such as membrane lipids. (**c**) Nanozymes possessing peroxidase- or oxidase-like activity play a pivotal role in disrupting the membrane structure and degrading the biofilm matrix, thereby exerting antibacterial and antibiofilm effects. This process ultimately results in bacterial cell death. Reproduced with permission from ref [88]. Copyright 2022 MDPI

In addition to the peroxidase-like activity, which has the potential to boost the antibacterial efficacy, oxidase-like activity also plays an equally significant role in the mechanism of how antibacterial agents work. Oxidase is an essential enzyme that can catalyze the redox reaction in which oxygen is involved. Activation of O_2_ by metals is required for oxidase-like activities that metals can carry out. Molecular oxygen (O_2_) is oxidized and transformed to either water (H_2_O) and oxygen or hydrogen peroxide (H_2_O_2_) through reactions that are catalyzed by oxidases (in some cases to superoxide radicals, O_2_^−^). During the catalytic process, active free radicals are created, which have an antimicrobial effect on the environment. H_2_O_2_ can directly oxidize the outer structure of bacteria due to its high degree of reactivity. This causes the permeability barrier of the bacteria to be destroyed, as well as a disruption of the electrochemical balance between the internal and external substances of the bacteria, ultimately resulting in bacterial death. Similarly, O_2_ can directly react with many biomolecules, such as proteins, nucleic acids, and more, ultimately resulting in bacterial death. In general, oxidase- and peroxidase-like activities comprise the primary mechanisms of antimicrobial efficacy for metal NCs [88, 92]. Figure 5 shows catalytic reactions and antibacterial mechanisms of NCs with peroxidase- or oxidase-like enzyme activities. Figure 5a shows NCs with peroxidase-like activity catalyzing the reduction of H_2_O_2_ and producing free ⋅OH. NCs with oxidase-like activity catalyze O_2_–^1^O_2_ or even to single oxygen atoms, as shown in Fig. 5b. Both ⋅OH and ^1^O_2_ are strong oxidants that convert the substrate (S), such as membrane lipids, to ox-substrate (Sox). Figure 5c shows NCs with peroxidase- or oxidase-like activity that can disrupt membrane structures or degrade the biofilm matrix, ultimately killing the bacteria [88].

Zheng et al. produced AuNCs using four distinct forms of mercaptopyrimidine as ligands. These mercaptopyrimidine forms were 4-amino-2-mercaptopyrimidine (AMP), 4,6-diamino-2-mercaptopyrimidine (DAMP), 4-amino-6-hydroxyl-2-mercaptopyrimidine (AHMP), and 4,6-dihydroxyl-2-mercaptompyrimidine (DHMP). According to the findings, AuNCs-DAMP demonstrated the most potent antibiotic activity with the lowest minimum inhibitory concentration (MIC) against the proliferation of methicillin-resistant *S. aureus* (MRSA), vancomycin-resistant *Enterococcus* (VRE), and multidrug-resistant (MDR) strains of *Klebsiella pneumonia*, *Acinetobacter baumannii*, *Pseudomonas aeruginosa*, and *E. coli*. In contrast, the bactericidal effects of AuAMP, AuAHMP, and AuDHMP against clinical ESKAPE (*Enterococcus faecium*, *S. aureus, K. pneumoniae, A. baumannii, P. aeruginosa, and Enterobacter* spp.) groups were not particularly strong. Comparisons were made between AuNCs and AuNPs of larger sizes (ca. 6 nm) to investigate the influence of particle size on the antibacterial activity. Compared to AuNPs, the formation of ROS significantly increased with the use of AuNCs-DAMP. They postulated that the oxidase-like and peroxidase-like properties of AuNCs could cause ROS formation, whereas the oxidase-like and peroxidase-like capabilities of AuNPs could not [93].

### Nano-bio interface interactions of NCs

**Fig. 6:**
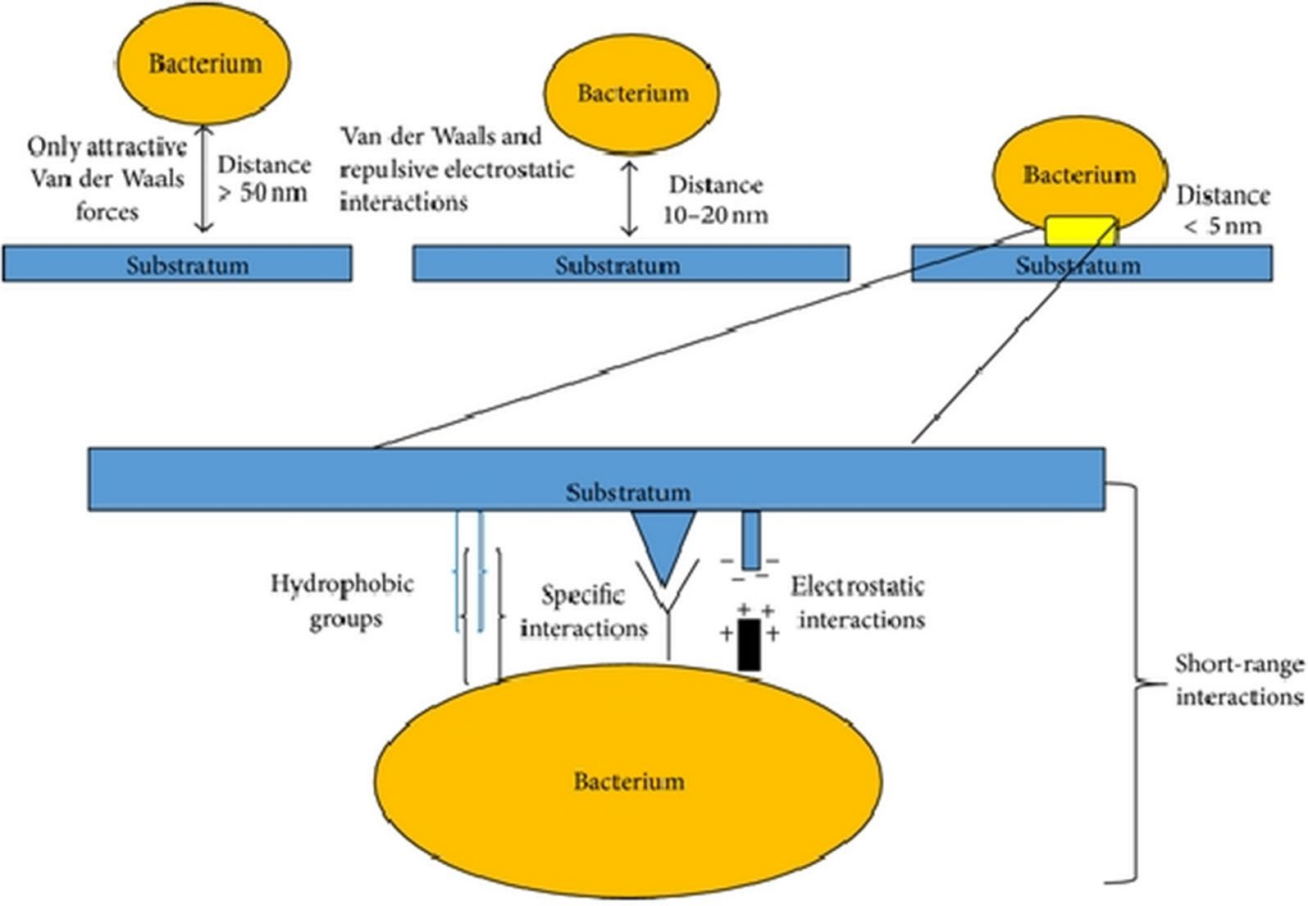
Phase one of bacterial adhesion. Reproduced with permission from ref [95]. Copyright 2014 Hindawi

Metal NCs are extremely useful for achieving antibacterial effects and combating internal germs due to their small dimensions. In general, metal NCs are capable of interacting with the cell walls of bacteria by a wide range of processes, some of which include electrostatic interactions, van der Waals (VDW) forces, receptor-ligand interactions, and hydrophobic interactions, among other mechanisms [94]. Bacterial adhesion is a process that occurs during the creation of bacterial biofilms. This process was shown to influence the degree to which an individual organism is susceptible or resistant to standard antibiotics. Studies showed that the size of metal nanocrystals can effectively demonstrate the influence of antibacterial activity [39]. Bacterial adhesion to material surfaces is a two-stage process. The first stage is an initial, immediate, and reversible phase of physical interaction. The second stage is a time-dependent and irreversible molecular and cellular interaction phase. The initial attraction of cells to the surface occurs during the first phase of bacterial adhesion. The influence of physical forces such as Brownian motion, VDW forces, gravitational forces, the effect of electrostatic charges, and hydrophobic interactions causes the initial attraction. The first stage of bacterial adhesion is characterized by the initial attraction of a cell to the surface, which occurs due to the action of physical forces. Figure 6 depicts the various types of physical interactions, which can be further broken down into long-range interactions (which are nonspecific and take place at more than 50 nm between cells and the surface) and short-range interactions (which take place at a distance of less than 5 nm and involve ionic and dipole interactions, hydrophobic interactions, and hydrogen bonding). The second phase involves molecular-specific reactions between the structure of the bacterial surface and the surface of the substratum, either uncoated or coated with host matrix proteins, while the first phase involves the formation of a biofilm (including such substances as albumin, vitronectin, fibronectin, fibrinogen, and laminin) [95].

In studying the antibacterial effect of NCs, it is essential to pay attention to the kind of cell membrane present before considering NCs that interact with biomembranes. This is because different cell membranes are associated with different types of bacteria. Proteins and lipids make up the majority of the membrane’s composition. The amphiphilic organic molecules and lipids that make up a typical cell membrane are the fundamental building blocks of the membrane. In general, lipids comprise two significant components: lengthy hydrophobic tails and a relatively compact hydrophilic head. Liposomes, spherical and cylindrical micelles, bilayer membranes, and other structures are compositions contained in lipids that make their structures stable in water. Hydrophobic interactions between the lipid tails on the inside of the membrane and interactions of the head groups with water and each other on the surface contribute to the stability of the lipid bilayer. The lipid bilayer is the fundamental component of the cell membrane’s structure.

**Fig. 7:**
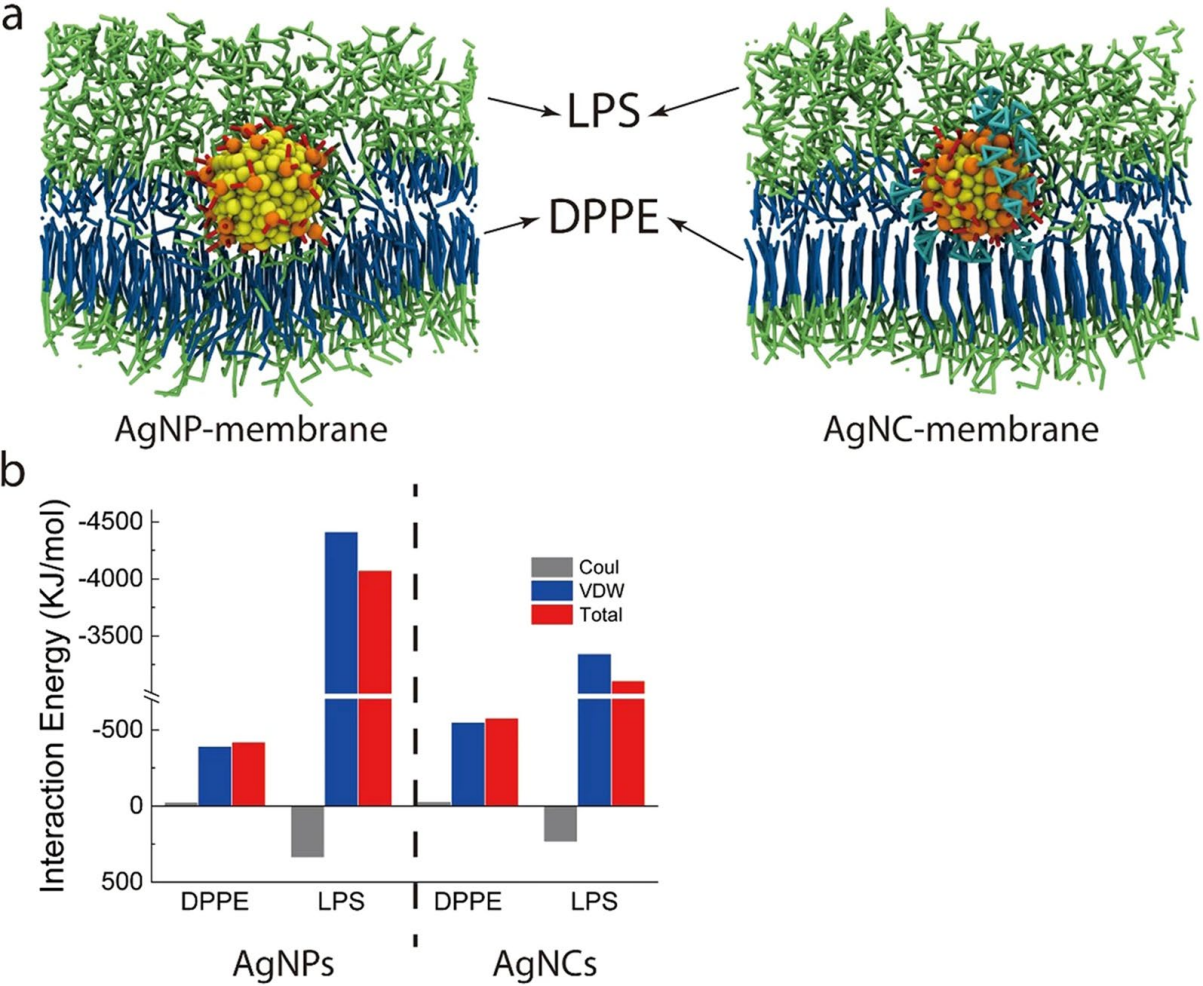
Interactions of silver nanoparticles (AgNPs) and silver nanoclusters (AgNCs) with bacterial membranes using a coarse-grained molecular dynamics (CGMD) simulation method. Reproduced with permission from ref [99]. Copyright 2020 Nature Publishing Group

The size of the NCs can directly impact interactions of NCs with their nano-bio interface. Computer simulation studies are one approach utilized to determine the effect that size has on interactions between NCs and cell membranes. The use of simulations can provide atomic-level accuracy about dynamic structural changes, which can reveal the mechanism of nano-bio interface interactions, which can be accomplished through the use of feasible modulation. Endocytosis can be achieved in many ways, depending on the size of the NCs [96]. Gupta et al. carried out coarse-grained molecular dynamics (CGMD) to assess the penetration of different-sized AuNPs (2–5 nm) via a lipid membrane model found in the skin. Due to their higher diffusivity, smaller AuNPs could enter the interior of the bilayer more quickly than their larger counterparts. The computation of the permeation-free energy is shown if, in the head group of the bilayer, there is a minor barrier to neutral hydrophobic AuNPs, and the permeation-free energy is at its lowest for charged AuNPs. The permeability was at its highest when neutral 2-nm AuNCs were present, and it was at its lowest when cationic 3-nm AuNPs were present [97]. According to findings of Yue et al., size dependence is responsible for the uptake of NPs by cellular membranes. Using computer simulation techniques, they were able to demonstrate that the process of internalization of NPs of varying sizes is a cooperative one. The combined effect, interpreted as a result of membrane curvature meditation, is impacted by the semi-membrane tension and the concentration of NPs on the membranes. At the same time, NPs of intermediate size tended to aggregate into a linear pearl-chain-like arrangement, and large-sized NPs likely detached from each other and were separately internalized. In general, NCs cluster in a closely packed agglomeration on the membrane and are internalized [98]. In addition, Chen et al. carried out CGMD simulations to focus primarily on distinctions between the methods by which AgNPs and AgNCs penetrate bacterial membranes. It was assumed for the simulation that the outermost layer of the bacterial membrane is composed of LPS and dipalmitoyl-phosphatidylethanolamine (DPPE). They did this by applying an external force to the membrane, which allowed either AgNPs or AgNCs to pass through and reach the inside of the membrane. Then, they compared energy interactions that these NPs had with DPPE and LPS molecules as shown in Fig. 7 [99].

The first interaction of AgNPs and AgNCs with bacterial membrane structures is shown in Fig. 7a. The head groups of LPS and DPPE are represented by green sticks, while blue sticks represent the tails of LPS and DPPE in this illustration. Sticks representing the carboxyl groups and adamantanes are respectively colored red and cyan. Yellow and orange beads respectively represent silver and sulfur atoms that make up the core. Figure 7b illustrates energy interactions, where the energy exchange is depicted. VDW force interaction energy of AgNPs and AgNCs with LPS primarily determines their relative translocation. This is the case for both types of particles. Because of the hydrophilicity of AgNPs, the contact of VDW forces with the hydrophobic tails of DPPE is weaker, and interactions between the polar heads of LPS aree stronger than those of amphiphilic AgNCs. Both of these results are in contrast to the amphiphilicity of AgNCs. The total interaction energy, which represents the attractive interaction of AgNPs and AgNCs with the molecules, is depicted as a negative number in the diagram located above. This number indicates a stronger binding of AgNPs and AgNCs with the molecules. Conversely, a positive number represents the repulsive interaction. As a result, AgNPs have a more-significant energy barrier to overcome before they can successfully permeate the membrane. Compared to AgNCs, AgNPs are more likely to be driven away by the negatively charged heads of LPS. Consequently, the amphiphilic characteristic of AgNCs with two ligands can considerably lessen energy interactions with LPS, which more readily allows the breaching of the outer membrane [99]. In Table 1, we summarize several examples of antibacterial applications according to NC size covered in this review.

**Table 1:** Effects of nanocluster size on antibacterial applications

Material	Target pathogens		Nanocluster size	Result	Antibacterial mechanism	References
	Gram-positive bacteria	Gram-negative bacteria				
Gold nanoclusters (AuNCs)-6-mercaptohexanoic acid (MHA); gold nanoparticles (AuNPs)	*Staphylococcus aureus* and *Bacillus subtilis*	*Escherichia coli* and *Acinetobacter baumannii*	AuNCs = 2.33 ± 0.90 nm; AuNPs = 7.08 ± 1.5 nm	Compared to larger NPs, AuNCs-MHA have a considerably higher surface area to volume ratio	They may also increase DNA damage, membrane damage, reactive oxygen species (ROS) generation, and metabolic inactivation	[76]
AuNCs-3-mercaptobenzoic acid (MBA)	*S. aureus*	*E. coli*	AuNCs = < 2 nm Au NPs = 3 and 5 nm	AuNCs-MBA have a high antimicrobial efficacy because of their ultrasmall size	AuNCs of < 2 nm readily passed through ultrasmall pores of the bacteria cell wall, internalizing themselves, and killing the bacteria by simple diffusion	[61]
Luminescent copper nanocluster (CuNC)-doped hydroxyapatite (HAP) NPs	*S. aureus* MTCC 96 and *B. subtilis* MTCC 1305	Green fluorescent protein (GFP) expressing recombinant *E. coli*, *Pseudomonas aeruginosa* MTCC 2488, and *E. coli* DH5α	Doped HAP NPs = 32.64 ± 7 nm and Undoped HAP NPs = 24.0 ± 6.0 nm; CuNCs = 1.75 ± 0.3 nm CuNCs incorporated into doped HAP NPs = 1.71 ± 0.5 nm	Compared to NPs, CuNCs doped with HAP had a lower minimum inhibition concentration (MIC) and exhibited greater antimicrobial activity	Damage to bacterial cell membranes	[^100^]
AuNCs-MHA	S*. aureus, S. epidermidis, B. subtilis*	*E. coli, P. aeruginosa*	AuNCs = ca. 2 nm; AuNPs = 6.0 ± 3 nm	The improved antibacterial action of AuNCs was determined not just by the ligand identity or its density on the surface but rather by the ultrasmall AuNCs as a whole as an entity and compound. They have a high surface-to-volume ratio because of their extraordinarily diminutive size	Membrane internalization, metabolic imbalance, intracellular ROS production	[79]
Dpep-AgNCs; Opep-AgNPs	*S. aureus*	*E. coli* and *Shewanella oneidensis* MR-1	Dpep-AgNCs = 1.25 nm; Opep-AgNPs = ca. 5 nm	Due to the extra-small particles which can increase the potential to permeate into the bacteria, Dpep-AgNCs demonstrated greater antimicrobial activity than Opep-AgNPs	Electrostatic and van der Waals (VDW) forces enable their subsequent internalization	[80]
THPC-AuNPs; THPC-AuNPs/MTU; THPC-AuNPs/Prot; MTU-AuNCs; Prot/MTU-AuNCs	*S. aureus*	*E. coli*	Prot/MTU-AuNCs = ca. 1.5 nm; AuNPs = not mentioned	Prot/MTU-AuNCs interacted synergistically due to their distinct size and the bacteriostatic properties of the Prot capping shell. THPC-AuNPs caused negligible harm to the bacteria’s membrane cell	Membrane internalization, ROS production	[101]
AuDAMP; AuAMP; AuAHMP; AuDHMP;	*S. aureus* ATCC29213, methicillin-resistant *Staphylococcus aureus* (MRSA), Vancomycin-resistant *Enterococcus faecium* (VRE)	*E. coli* ATCC35218, multidrug-resistant (MDR) *A. baumannii*, MDR *E. coli*, MDR *P. aeruginosa*, MDR *K. pneumoniae*	AuAMP = 1.8 ± 0.7 nm; AuDAMP = 1.7 ± 0.5 nm; AuAHMP = 1.9 ± 0.71 nm; AuDHMP = 1.7 ± 0.2 nm; AuNPs-DAMP = ca. 6 nm	Due to their oxidase-like and peroxidase-like nature, AuNCs induced ROS production whereas AuNPs could not, which showed a greater antibacterial effect	Cell membrane destruction, DNA damage, ROS generation	[93]
AgNCs and AgNPs with mercaptosuccinic acid (MSA)	–	*P. aeruginosa, A. baumannii*, and *E. coli*	AgNCs = 2.8 nm; AgNPs = 3.8 and 3.2 nm	In contrast to AgNPs, which tended to self-aggregate outside of bacterial cells, AgNCs had a far higher degree of intracellular localization. Compared to amphiphilic AgNCs, VDW interactions between AgNPs and the hydrophobic tails of DPPE were significantly weaker. In contrast, VDW interactions between AgNPs and the polar heads of lipopolysaccharide (LPS) were significantly more robust. For AgNPs to be able to pass through the membrane, they had to overcome a significantly greater amount of energy	Membrane damage and ROS generation	[99]

DAMP, 4,6-diamino-2-mercaptopyrimidine; AMP, 4-amino-2-mercaptopyrimidine; AHMP, 4-amino-6-hydroxyl-2-mercaptopyrimidine; DHMP, 4,6-dihydroxyl-2-mercaptopyrimidine; DPPE, dipalmitoyl-phosphatidylethamolamine

## Overview of surface ligands of metal NCs in antibacterial applications

In addition to the cluster size (number of atoms), the nature of the protective ligands significantly impacts the stability, solubility, and optical characteristics of NCs. This alteration affects the entire cluster. Surface ligand engineering of NCs is one of the strategies for producing and determining the formation of NCs with well-controlled and adjustable physicochemical properties, such as optical absorption, hydrodynamic size, photoluminescence, and molecular chirality [102]. The number of metal atoms in metal NCs can range from one to hundreds, and they are surrounded by organic ligands that protect them layer by layer. Such organic ligands are typically used to inhibit the aggregation of metal NCs in solution. They also come in handy when it comes to isolating target NCs. In addition, the surface ligands’ design plays a significant role in determining the physical and chemical properties of ligand-protected metal NCs. The molecular formula for metal NCs, which can be represented as [M_n_L_m_]^q^, depicts NCs that have the same kinds of chemical and physical properties as molecules (M and L are the core metal atoms and the protecting ligands, while n and m are the numbers of core metal atoms and the number of protecting ligands, respectively, and q represents the charge possessed by these ligand-protected metal NCs) [102]. Several types of protecting ligands used for stabilized metal NCs include thiolates, phosphines, selenolates, tellurolates, and alkynes [103]. The design of surface ligands also plays an essential role in the physical and chemical properties of ligand-protected metal NCs. These properties include highest occupied molecular orbital (HOMO)-lowest unoccupied molecular orbital (LUMO) electronic transitions, photoluminescence, discrete redox behavior, intrinsic magnetism, and optical chirality [102]. As a crucial component that impacts both the molecular properties and performance behaviors of metal NCs, the design and choice of the type of protecting ligands used can also have effects, and this must be adjusted to the purpose of the metal NC application itself.

**Fig. 8:**
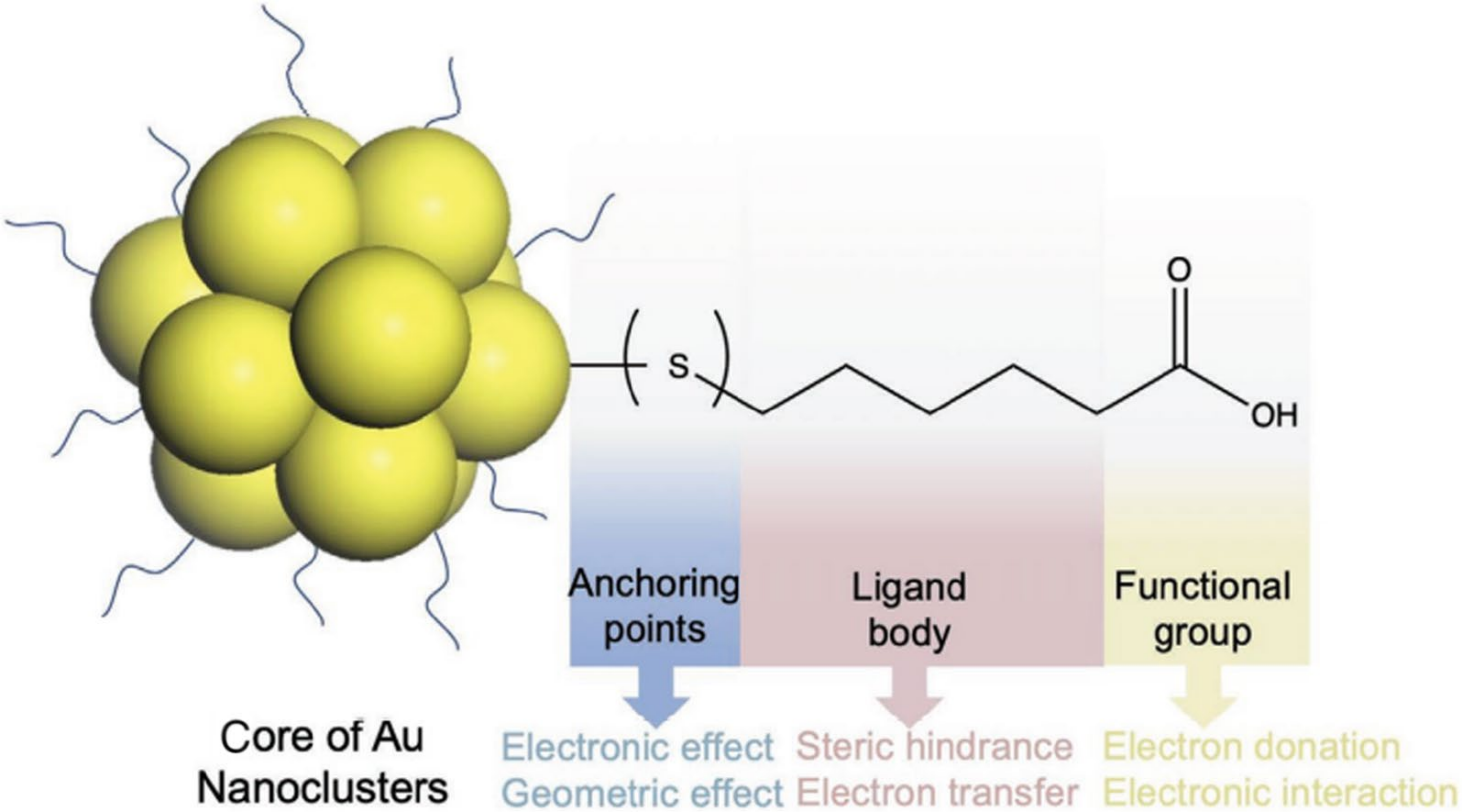
Schematic illustration of the three parts (anchoring point, ligand body, and functional group) of the protecting ligands on gold nanocluster (AuNC) surfaces with mercaptohexanoic acid (MHA) as a ligand model. Reproduced with permission from ref [102]. Copyright 2021 John Wiley and Sons

Figure 8 illustrates how metal NCs (Au was used in this example) with organic ligands on the surface can be separated into three distinct portions. First, the inner part is the anchoring point directly interacting with the metal atoms, enabling the ligands to create strong covalent bonds with the metal atoms on the NC surface. Because of this, the structure and physicochemical properties of metal NCs are significantly altered. Second is the ligands’ core, which can include things like benzene rings with or without alkyl substituents, alkyl chains of varying chain lengths, and other such things. In addition to conjugation effects, this section of the ligands also contains other molecular physical forces (such as hydrophobic and VDW interactions) between the ligands located on the NC surface. Through steric hindrance and electron transport, these forces affect the structures of the metal NCs and the physicochemical properties of the materials. The third part is the functional group on the surface ligands (most relevant to hydrophilic ligands), including carboxylic (–COOH) and amine (–NH_2_) groups. All three of the metal NCs’ surface ligands are significant when collectively considered since they function synergistically (as a whole) to define the chemical and physical characteristics of metal NCs. Due to the ultrasmall size of this type of NP, the impacts of surface ligands on metal NCs, which serve as the outermost layer, are much more noticeable. The performances of these ligands in various applications are governed by their direct contact with the external environment, which includes molecules, biomolecules, cells, and tissues [102].

### Electrostatic interactions and VDW force factors in bacterial attachment to metal NCs

**Fig. 9:**
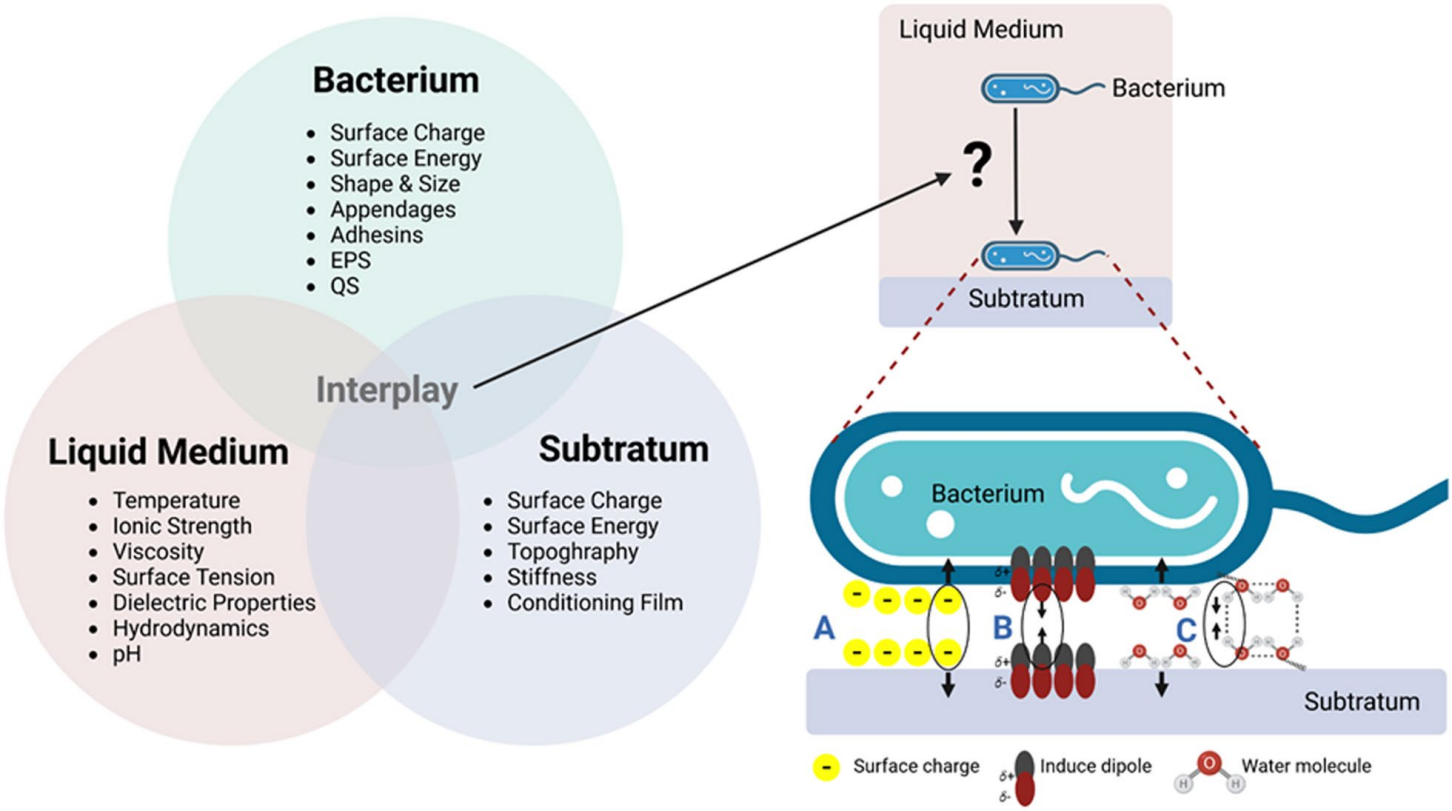
An illustration of initial attachment factors of a bacterium to a solid–liquid interface, the interplay between the bacterium’s properties with the solid surface and liquid medium, and physicochemical force probabilities between the bacterium and the solid surface that affect attachment. **A** Electrostatic interactions; **B** van der Waals interactions; **C** hydrophobic interactions. EPS, extracellular polymeric substances; QS, quorum sensing

For a bacterial cell to attach itself to a biotic or abiotic surface, it must first locate the surface, then approach it, and finally, sense how close it is to the surface. This is a complex and multistage process. In the case of motile bacteria, the active movement of the bacteria themselves may be the source of the transfer of bacterial cells into contact, in addition to the potential role played by physical processes such as diffusion (Brownian motion) and convective flow [104]. This can be explained by an illustration of the interplay and physicochemical forces among a solid surface, a bacterium, and the liquid medium as shown in Fig. 9. Bacterial adhesion is influenced by the way the bacterium interacts with the surface. Bonds that may be involved in the interaction between a bacterium and surfaces include: (1) electrostatic bonds, (2) hydrophobic bonds, (3) VDW bonds, (4) hydrogen bonds, (5) charge-transfer bonds, and (6) biospecific bonds. Biospecific bonds can never be separated and consist of one or more of the (1)–(5) bonds. The close spatial fit between groups involved in the bond is what causes the particular and frequently quite intense interactions. According to various studies, polysaccharides, proteins, and lipids—elements that make up the outer layer of bacteria—do not attach to chromatographic adsorbents via hydrogen bonds or charge-transfer processes, except in a very small number of unusual instances and then especially at low pH (except when specially designed columns are used). As a result, interactions between the surface and a bacterium are not centered on (4) and (5) type bonds. VDW forces are created by instantaneous dipoles in one atom which causes a dipole in an adjacent atom and produces a weak attractive force. Simple materials’ ability to stick together even when they lack electrostatic, dipole, or electron-sharing capabilities may be explained by this universal force [105]. VDW forces may become operative forces even if hydrophobic interactions and electrostatic repulsions play key roles in bacterial adherence. Thus, when molecular groups participating in hydrophobic bonds are sufficiently close to one another and produce short-range forces, hydrophobic bonds may be changed into or strengthened into VDW interactions.

Typically, a two-step model is used to describe how bacterial cells adhere to surfaces. Reversible attachment occurs in the first step, followed by irreversible attachment in the second step. The initial weak attachment forces between the bacterial cell and the surface include electrostatic forces, VDW forces, and hydrophobic interactions. Although bacterial cells are reversibly bound to a surface, shear forces like washing and turbulent fluid flow may quickly dislodge them. Electrostatic repulsion may sometimes be stronger than the aforementioned weak attractive forces [106]. As natural surfaces and bacteria are often negatively charged, this causes repellent electrostatic interactions that lead to irreversible adhesion [106, 107].

Due to the basic principle that bacteria are relatively large colloidal particles, surface chemistry and colloid theories provide a decent description of the behaviors of these colloidal particles [107]. Interfacial tensions among the fluid medium, bacteria, and solid substrate are used to characterize the shift in free energy when bacteria move from planktonic to surface-attached in the thermodynamic method. According to the Derjaguin-Landau-Verwey-Overbeek (DLVO) theory of bacteria, free energy results from an equilibrium between attractive Lifshitz-VDW forces and attractive or repulsive electrostatic forces. The expanded DLVO (xDLVO) method incorporates additional forces to explain "hydrophobic attractive" and "hydrophilic repulsive" interactions, such as Lewis acid–base and Brownian forces. Bacteria may also have adhesins, such as pili, fimbriae, flagella, LPS, and extracellular polymeric compounds, that allow them to penetrate the secondary energy maximum and irreversibly adhere in addition to these physicochemical interactions [108].

**Fig. 10:**
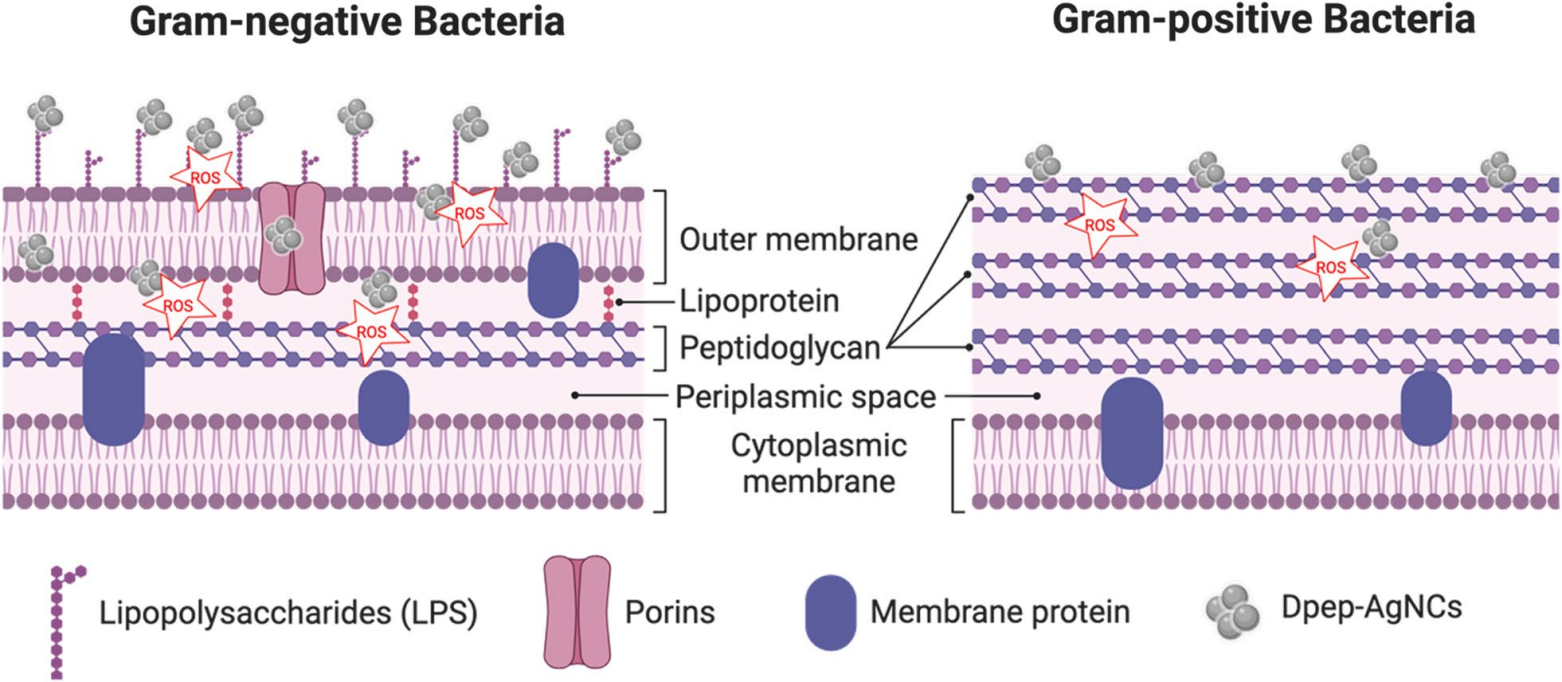
Gram-positive and gram-negative bacteria are combated using Dpep-silver nanoclusters (AgNCs) in the antibacterial assay. The Ag + ions that are internalized in both types of bacteria can induce reactive oxygen species (ROS) inside bacterial cells. Dpep-AgNCs internalize into gram-negative bacteria through interactions with lipopolysaccharides (LPS) and porins, while they enter gram-positive bacteria through interactions with peptidoglycan

Ye et al*.* reported the development of a synergistic antibacterial platform that included two unique Ag^+^ species of bactericides with well-designed antimicrobial peptides containing plentiful arginine and leucine residues (Dpep). To ensure that both Ag^+^ species and antimicrobial peptides are delivered in one package, Dpep was used as the capping ligand during the synthesis of AgNCs. Compared to the other NCs, the as-prepared Dpep-AgNCs had much greater antibacterial activity, which may be partly explained by their increased positive charges and stronger target binding to the LPS of gram-negative bacteria. Dpep-AgNCs performed better because of the increased local concentration of Ag^+^ ions and the greater antibacterial activity of the surface ligands. The initial interactions of the positively charged Dpep-AgNCs with the negatively charged membrane of bacteria may be facilitated by electrostatic and VDW forces, allowing for their further internalization. Additionally, the outer tails of the antimicrobial peptides attach to the negatively charged LPS and further penetrate the cell wall (Fig. 10) [80].

According to another report by Pang et al., electrostatic interactions between pyridinium groups on the surface of AuNCs and the wall teichoic acid (WTA) phosphonate groups on bacteria allow for the attachment of alkyl-thiolated zwitterionic and pyridinium ligand AuNCs to both gram-positive and gram-negative bacteria. The initial electrostatic interactions further encouraged widespread contact among the non-charged regions and increased their connections through VDW forces, which ultimately contributed to around 57% of the total interaction, when the cationic ligands engaged with the oppositely charged WTA layer. In the two types of bacteria, it was found that these AuNCs with ligands were more potent towards gram-positive strains than towards gram-negative ones. The different surface structures of gram-positive and gram-negative bacteria are the reason for this phenomenon. Gram-positive bacteria have a unique cell wall component called WTA, which is made up of repeated poly(glycerol phosphate) units with a phosphodiester terminal. The cell membrane is extremely negatively charged and open to the binding of cationic molecules, since WTA makes up the polyanionic network. Additionally, gram-positive bacterial membranes include around 80% anionic lipids, compared to just 30% in gram-negative strains, which include phosphatidylglycerol (PG) and cardiolipin. Furthermore, unlike human cells, which only have negatively charged phospholipids on the interior layer, bacterial cell membranes have negatively charged phospholipids on both sides. Some positively charged antibacterial drugs may be able to distinguish between mammalian cells and bacteria based on this differential [109].

#### Approach bacterial geometrical shape

**Fig. 11:**
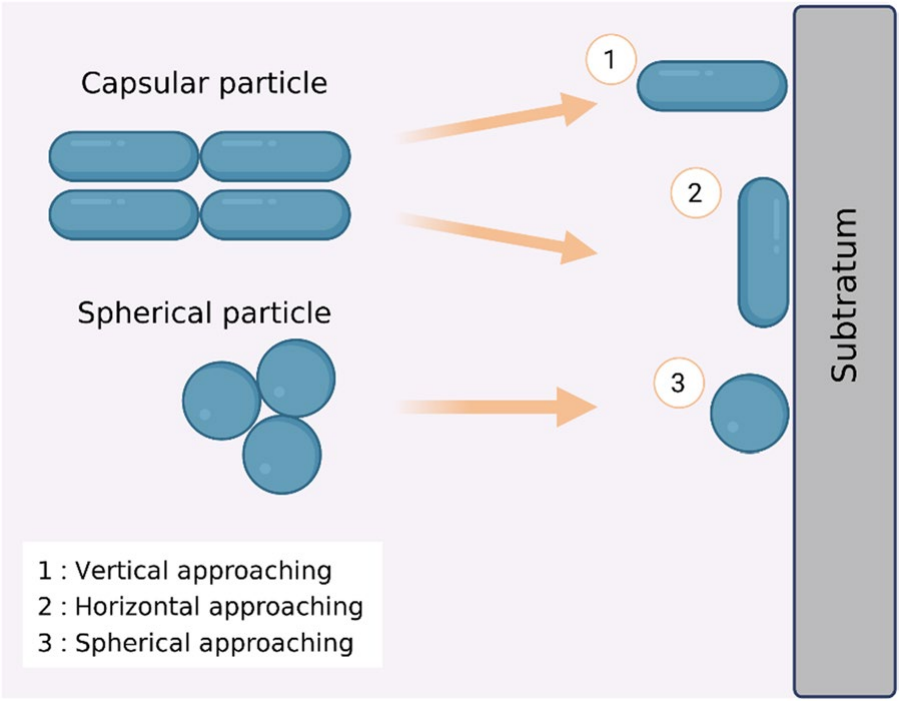
Illustration of a bacterium's geometric shape when approaching the surface of a substratum

One factor that can affect the VDW interaction potential and energy is different approach shape profiles of bacteria attached to the material. This was explained by Fathiah et al., who measured and obtained the profile of the VDW interaction potential on the geometrical shape of bacteria attached to the material using Hamaker’s microscopic numerical approach. The bacterial approach profile used was a capsular particle (vertical and horizontal) and spherical particle which was made as an approach for the two types of capsular particle attachments (Fig. 11). According to general scanning electron microscopy (SEM) and other microscopic investigations of bacteria on surfaces, a capsule-shaped bacterium will most likely be in a horizontal position when it approaches the surface. The VDW interaction potential becomes more negative (indicative of an attractive interaction between the bacterium and the mineral surface) as the bacterium moves closer to the mineral surface. This suggests that the bacterium and the mineral surface are positively interacting. The numerical results revealed that a capsule-shaped bacteria horizontally approaching a mineral surface was more appealing than one vertically approaching it. This was the case regardless of which direction the bacterium approached the surface. On the other hand, the VDW interaction potential of the spherical shell-sphere was significantly more enticing when contrasted with the other three cases (more negative). This was because of factors such as differences in density among bacterial shapes (cylinders, capsules, and spheres), which require different energy requirements to allow them to adhere to surface material, the type of mineral surface that was used, and the geometrical shape and curvature effect of the bacterium, which affect the settlement of the bacterium onto the material surface. These factors all play a role in determining how a bacterium settles onto a material’s surface [110].

#### Surface topography

It was shown that the surface topography has a major influence on bacterial adhesion, and different mechanisms seem to be more dominant at micro- and nano-scales. Physicochemical interactions between bacteria and surfaces play important roles in bacterial attachment. However, defining a comprehensive physicochemical theory of bacterial attachment is very difficult due to the chemical, biological, and structural complexity of bacterial cells that are frequently combined with complex substrate conditions. According to the traditional Derjaguin-Landau-Verwey-Overbeek (DLVO) theory, the sum of electrostatic (EL) and Lifshitz-van der Waals (LW) interactions produces attractive forces that determine the overall interaction force between a bacterial cell and a surface. Because bacterial cells and natural surfaces are usually negatively charged in an aqueous medium, the repulsive electrostatic energy is strengthened when the ionic strength of the aqueous medium is weakened. When a bacterial cell approaches a surface in an aqueous medium with low ionic strength it encounters an energy barrier that is insurmountable by motility or Brownian motion alone. On the other hand, when the ionic strength of the medium is high, this energy barrier disappears, making it possible for bacterial cells to approach the surface and easily irreversibly adhere to it. LW contacts are typically attractive, whereas EL interactions can either be attractive or repulsive, depending on the electrical charges of the bacterium and surface [104].

**Fig. 12:**
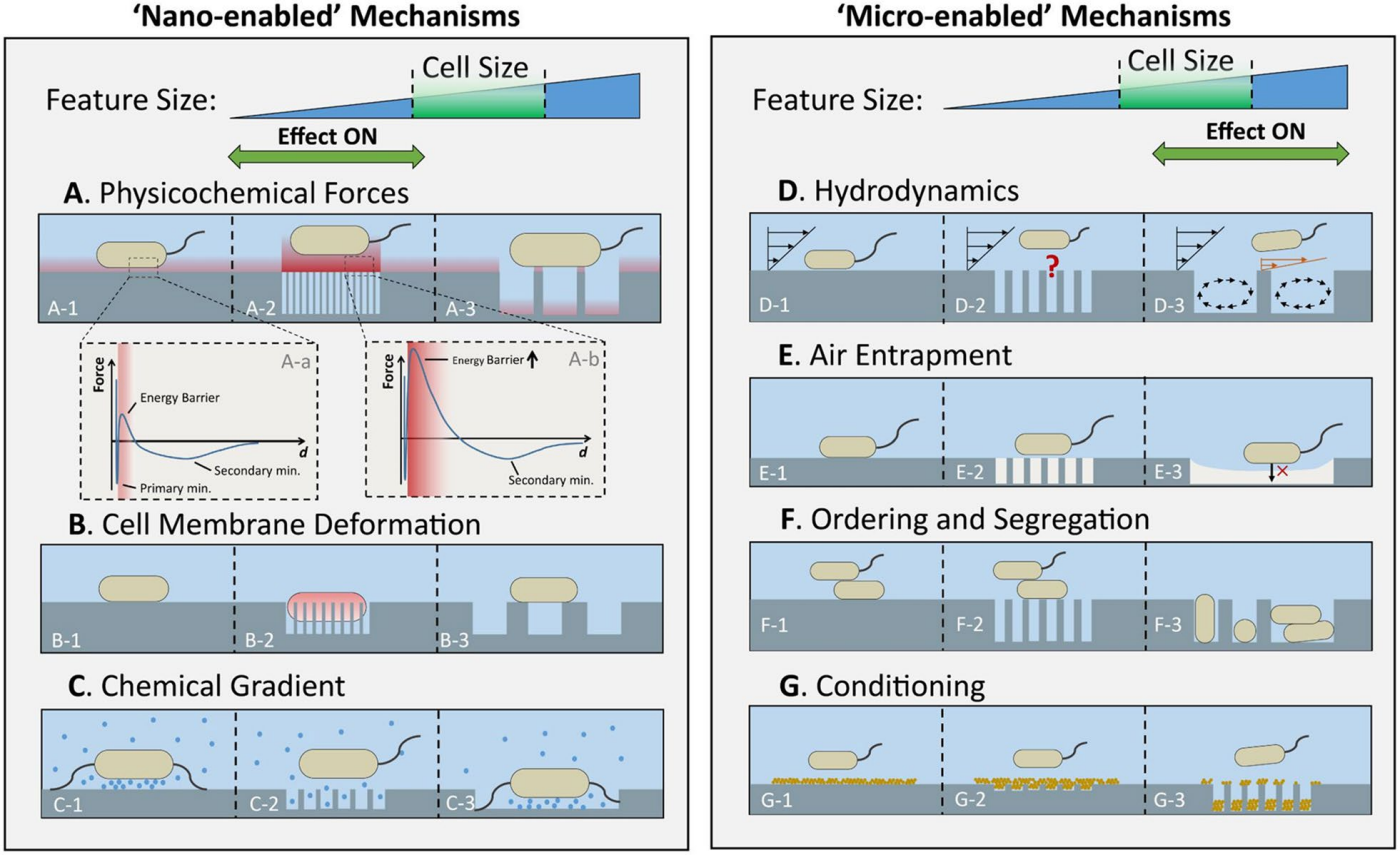
Effect of different topographies of the substratum surface on the anti-attachment effects of physicochemical forces of bacteria. Copyright 2019 Frontiers Media S.A. [104] All right reserved

Cheng et al. described a typical force-distance curve for bacterial interactions with surfaces that included two crucial characteristics: a peak signifying the energy barrier and a secondary minimum (Fig. 12). The energy barrier functions to prevent a trial attachment to other variations of bacteria by "blocking" them when they approach the surface. The secondary minimum is used as a representative of the movement limitation of bacteria in trapping cells in an energy well. Bacterial cells do not have the ability to overcome the energy barrier only by relying on their motility or Brownian motion, but their surface appendages can penetrate the energy barrier because of their small radius, and also can form a bridge from bacterial cells to their substrate. Through experimentation, total internal reflection microscopy has revealed this phenomenon [104].

Derjaguin’s integration and surface element integration (SEI) methodology are two popular ways to merge the influence of topography in particle-substratum interactions. Cheng et al. also explained that textured surfaces with equal chemical characteristics have lower interaction energy levels than their smooth equivalents. A surface with small pore sizes (15 and 25 nm) considerably reduced bacterial attachment compared to a smooth surface. In contrast to a smooth surface and surfaces with small pores, a surface with a larger pore size (50 nm) boosted bacterial attachment. Compared to surfaces with small pore sizes or smooth surfaces, surfaces with larger pore sizes imposed lower energy barriers on bacteria because the bacteria’s smaller external surface area led to significantly weaker interactions. This was the case regardless of whether the surface was smooth or had larger pore sizes [104].

### Surface charge factors in bacterial attachment to metal NCs

Surface chemistry, one of the physiochemical characteristics of metal NCs, may affect their antimicrobial capabilities. In addition to increasing the surface area by shrinking the NP size, it is also possible to modify the surface chemistry by altering the surface charge of metal NCs, which has the potential to significantly impact their antibacterial behavior [23]. By adjusting the surface ligands on metal NCs, the surface charge itself can be changed. Advanced surface chemistry can modify the type and makeup of protective ligands on the surface of metal NCs, producing a variety of ligands with the exact size, composition, and intended surface attributes [111]. Zheng et al. developed four types of cationic charge-conjugated AuNCs using ligands from mercaptopyrimidine derivatives: 4,6-diamino-2-mercaptopyrimidine (DAMP), 4-amino-2-mercaptopyrimidine (AMP), 4-amino-6-hydroxyl-2-mercaptopyrimidine (AHMP), and 4,6-dihydroxyl-2-mercaptopyrimidine (DHMP). Results of zeta potential measurements showed that these ligands mostly caused a positive surface charge on AuNCs with respective values for AuDAMP, AuAMP, AuAHMP, and AuDHMP of + 37.6 ± 1.1, + 33.6 ± 1.4, + 12.7 ± 0.7, and − 38.6 ± 1.8 mV. These materials were tested on gram-positive bacteria (*S. aureus*), gram-negative bacteria (*E. coli*), and ESKAPE superbugs (i.e., MDR *A. baumannii*, MDR *P. aeruginosa*, MDR *K. pneumoniae*, MDR *E. species*, methicillin-resistant *Staphylococcus aureus* (MRSA), and vancomycin-resistant *Enterococcus faecium* (VRE)). The minimum inhibitory concentration (MIC) required for each bacterial species was used to determine the antimicrobial activity of these mercaptopyrimidine-conjugated AuNCs. As a result, AuDAMP showed the strongest antibacterial effect compared to the other three types. In contrast, AuAMP, AuAHMP, and AuDHMP showed weak antibacterial effects, especially AuDHMP which had a negative surface charge [93]. These results are in excellent accordance with those of Tang et al. who explained that antibacterial agents with positive surface charges are thought to result in greater antibacterial potency [112]. The reason is that the strong positive charge on the surface of AuDAMP allows the promotion of electrostatic adsorption onto bacterial surfaces, resulting in the effective internalization of AuDAMP into bacteria [93]. Xie et al. also reported this with their analysis of the development of a cationic charge of AuNCs. The AuNCs which were conjugated with three types of positive ligands (i.e., quaternary ammonia (QA), nona-oligoarginine peptide (R9), and transactivator of transcription peptide (TAT)) revealed that ligand functionalization had a significant influence on the AuNCs’ zeta potential, which progressively shifted from negative to positive. These materials were shown to have antibacterial effects against gram-positive bacteria (*S. aureus* and MRSA) and gram-negative bacteria (*E. coli* and MDR *E. coli*) [113].

**Fig. 13:**
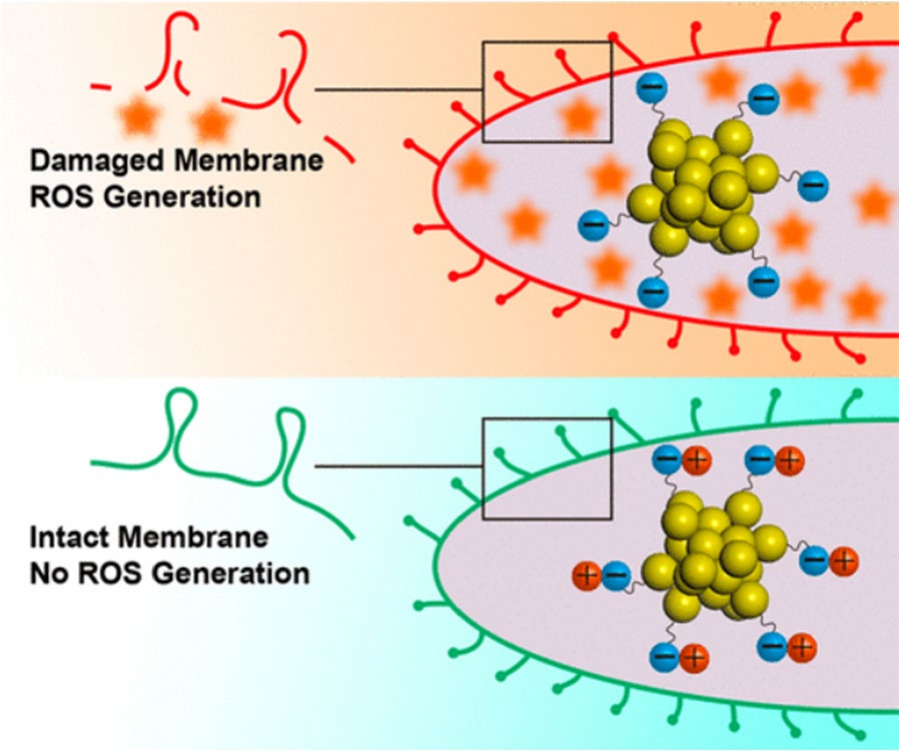
Schematic illustration of how the surface ligand chemistry of gold nanoclusters (AuNCs) determines their antimicrobial ability. Reproduced with permission from ref [111]. Copyright 2018 American Chemical Society

However, Zheng et al. had contrary results from this well-known paradigm, in which antimicrobial agents with stronger positive charges were more effective in killing bacteria. In that study, surface ligands on Au_25_(SR)_18_ (SR refers to thiolate ligands) were tuned at the atomic level to produce a series of AuNCs with the same Au atom numbers but with various surface characteristics. These AuNCs were designed to have a negative surface charge. The ligands (6-mercaptohexanoic acid (MHA), 3-mercaptobenzoic acid (MBA), L-cysteine (Cys), cysteamine hydrochloride (Cystm), and 2-mercaptoethanol (MetH)) were used to produce variations of one functional group ligand (Au_25_MHA_18_NCs, Au_25_Cys_18_NCs, Au_25_MBA_18_NCs) and two functional groups or bi-ligands (Au_25_Cystm_x_MHA_18-x_NCs and Au_25_MetH_x_MHA_18-x_NCs). Results demonstrated that the more-negatively charged Au_25_(SR)_18_ generated more ROS, improving the bacterial killing effectiveness as shown in Fig. 13 [111]. The positively charge groups could initiate interactions with bacterial cells that allowed them to insert their hydrophobic tail into the bacterial cell wall. This caused damage to the bacterial cell, ultimately leading to its death. As to the killing processes, this is one way how positive-surface AuNCs and negative-surface AuNCs are differentiated. However, producing ROS to kill bacteria is the primary mode of action of negatively charged surfaces. These data suggest that the positively charged groups added to the surface of Au_25_NCs may help with the initial engagement with bacterial cells but may ruin the Au_25_NCs’ capacity to produce ROS. This surprising outcome demonstrates the complexity of nano-bio interactions and may provide ideas for the development of high-performance AuNC-based antibacterial medications.

### ROS generation level on the bacterial killing mechanism by metal NCs

Metal homeostasis and redox balance are two examples of the many internal balances that bacteria must govern as an organism to keep themselves functioning. To kill bacteria, practical sterilizing methods used involve disrupting the bacteria's internal balance and increasing imbalances in their internal state [114]. ROS refer to a broad category of chemical compounds that are produced when oxygen is incompletely reduced, including hydrogen peroxide, superoxide anions, hydroxyl radicals, and singlet oxygen. When present in normal amounts, ROS are crucial for controlling a variety of physiological processes of the life cycle. However, an accumulation of ROS causes damaging oxidative stress, which may affect organisms in a variety of ways, particularly when intracellular reducing chemicals like protein thiols are consumed [23]. By producing ROS, which may disrupt normal bacterial metabolism and consequently kill bacterial cells, AuNCs are able to achieve antibacterial abilities. The formation of intracellular ROS in cells can also be modulated by organic ligands on the surface of materials. According to one study, various surface ligands on AuNCs induced the generation of different ROS, which had diverse antimicrobial killing effects.

Differences in ROS production resulting from different redox responses toward Au_25_NCs with different ligands were observed by Zheng et al. [111] By varying one functional ligand conjugated to AuNCs, Au_25_MHA_18_NCs and Au_25_MBA_18_NCs generated higher ROS levels, compared to Au_25_Cys_18_NCs which barely showed any change in ROS levels. As for bi-ligand-conjugated AuNCs, Au_25_Cystm_x_MHA_18-x_NCs and Au_25_MetH_x_MHA_18-x_NCs, also well generated ROS levels, similar to Au_25_MHA_18_NCs and Au_25_MBA_18_NCs. These differences in ROS generation can be explained by the different functional groups possessed by each ligand. Each of the MHA and MBA ligands only contains one carboxylic group, –COOH. While the Cys ligand contains one carboxylic and one amine group, –COOH and –NH_2_. Bi-ligand combinations of Cystm-MHA contain one carboxylic group –COOH from MHA and one amine group –NH_2_ from Cystm. The bi-ligand combination of MetH-MHA also contains one carboxylic group, –COOH, from MHA but also one hydroxyl group, –OH, from MetH. The reduced ROS production level by Au_25_Cys_18_NCs may be caused by the redox-buffering capacity of the conjugated functional groups. Positively charged –NH_2_ molecules have the ability to quench singlet oxygen; hence their presence reduces the amount of ROS generated. As a result, ROS generation further decreased the more positively charged –NH_2_ moieties that were added to the surface, decreasing the total ROS bioavailability and reducing the antibacterial effect. Contrary to positively charged groups, negatively charged groups which have –COOH groups produced better ROS, thus supporting a better antibacterial performance by Au_25_NCs conjugated by this group compared to other surface groups. This also explains why bi-ligand functional groups can well generate ROS, because these groups have a higher ratio of –COOH groups on the surface of their Au_25_NCs. Therefore, when the –COOH group was buffered with a neutrally charged –OH group, the ROS generation ability decreased, as in NCs with the MetH ligand which has the –OH functional group in its structure.

**Fig. 14:**
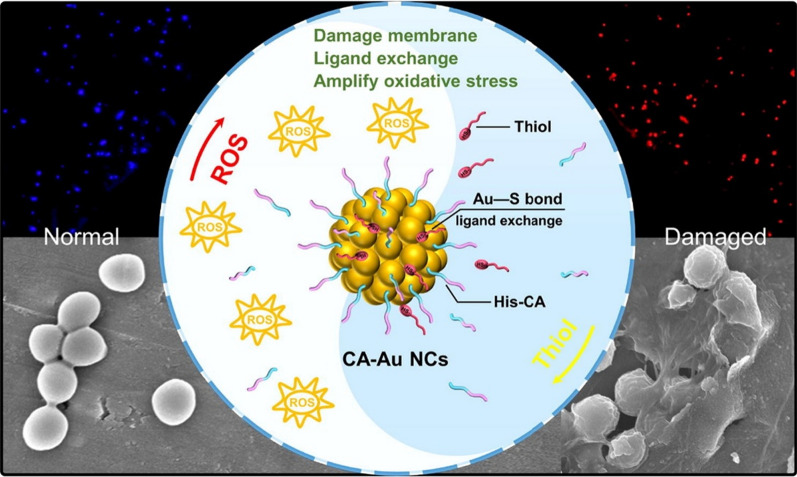
Schematic illustration of reactive oxygen species (ROS) generation of cinnamaldehyde (CA)-gold nanoclusters (AuNCs). Reproduced with permission from ref [114]. Copyright 2021 Elsevier

A recent study carried out by Meng et al. made efficient oxidative stress amplifier-conjugated AuNCs using histidine (His) as a stabilizer and cinnamaldehyde (CA) as a ligand with a ligand-exchange strategy. When ligands have a greater affinity for Au than stabilizing ligands of AuNCs, ligand-exchange reactions of AuNCs can be successful. The capacity of ultrasmall AuNCs to produce ROS increased by functionalization of CA on the surface of the AuNCs. The redox balance of oxidizing and reducing species in bacterial cells is significantly maintained by low-molecular-weight thiols of the bacteria. Thiol depletion results in a prompt accumulation of ROS in bacterial cells, which play a role in antibacterial effects. Additionally, His-CA groups on the surface were encouraged to exchange ligands with thiol species due to the strong Au-sulfur interactions between the CA-AuNCs and thiols. Therefore, increased ROS production and considerable thiol depletion result in magnified oxidative stress in bacteria, which ultimately resulted in bacterial mortality by disrupting the redox equilibrium of cells as shown in Fig. 14 [114].

### Regulating gene expressions by metal NCs

In line with how ligands on metal NCs regulate ROS generation, ligands conjugated to metal NCs can similarly control how genes are expressed by bacterial cells. For example, Zheng et al. demonstrated how ligand-conjugated AuNCs (Au_25_MHA_18_NCs, Au_25_MBA_18_NCs, Au_25_Cys_18_NCs, Au_25_Cystm_x_MHA_18-x_NCs, and Au_25_MetH_x_MHA_18-x_NCs) increased expressions of the *dmpI*, *narJ*, and *narK* genes, which are indicators of maintaining an intracellular redox equilibrium. There was excellent agreement between gene expression results from ligands with each type of functional group and ROS generation levels. This showed that surface ligands on Au_25_NCs modulate pro-oxidative enzymes to stimulate the formation of ROS inside bacterial cells. Similar to results of the ROS formation level, each type of functional group applied to the surface of AuNCs had an impact on changes in gene expressions at this level [111].

Differences in gene expressions of metal NCs due to the influence of ligands also occurred in a report by Zheng et al. who developed AuNCs with two ligands with different functional groups, p-mercaptobenzoic acid (Au-MBA NCs) and glutathione (Au-SG NCs). That study employed one reduction-regulating gene (*sod*) and three oxidation-regulating genes (*dmpI*, *narJ*, and *nark*). Toluene, o-xylene, 3-ethyl toluene, and 1,2,4-trimethylbenzene were oxidatively catabolized into relevant intermediates in the citric acid cycle by 4-oxalocrotonate tautomerase, which is encoded by the *dmpI* gene, and which produces ROS as a byproduct. The *narJ* and *narK* genes play key roles in respiratory nitrate reductase, which helps with nitrate transport and nitrate reduction by transferring electrons from nicotinamide adenine dinucleotide (NADH) or NADH phosphate (NADPH) to nitrate. The intracellular ROS scavenger, SOD enzyme, which has the potential to limit the oxidative metabolic process, is encoded by the reduction-regulating *sod* gene. According to data, Au-MBA NCs significantly increased expressions of pro-oxidative genes and significantly decreased expressions of antioxidative genes, but Au-SG NCs did not exhibit any discernible alterations. These gene expression findings further implied that the internalized AuNCs could lead to a metabolic imbalance by promoting pro-oxidative enzymes and suppressing antioxidative enzymes, which could cause the accumulation of intracellular ROS leading to bacterial death [61].

**Fig. 15:**
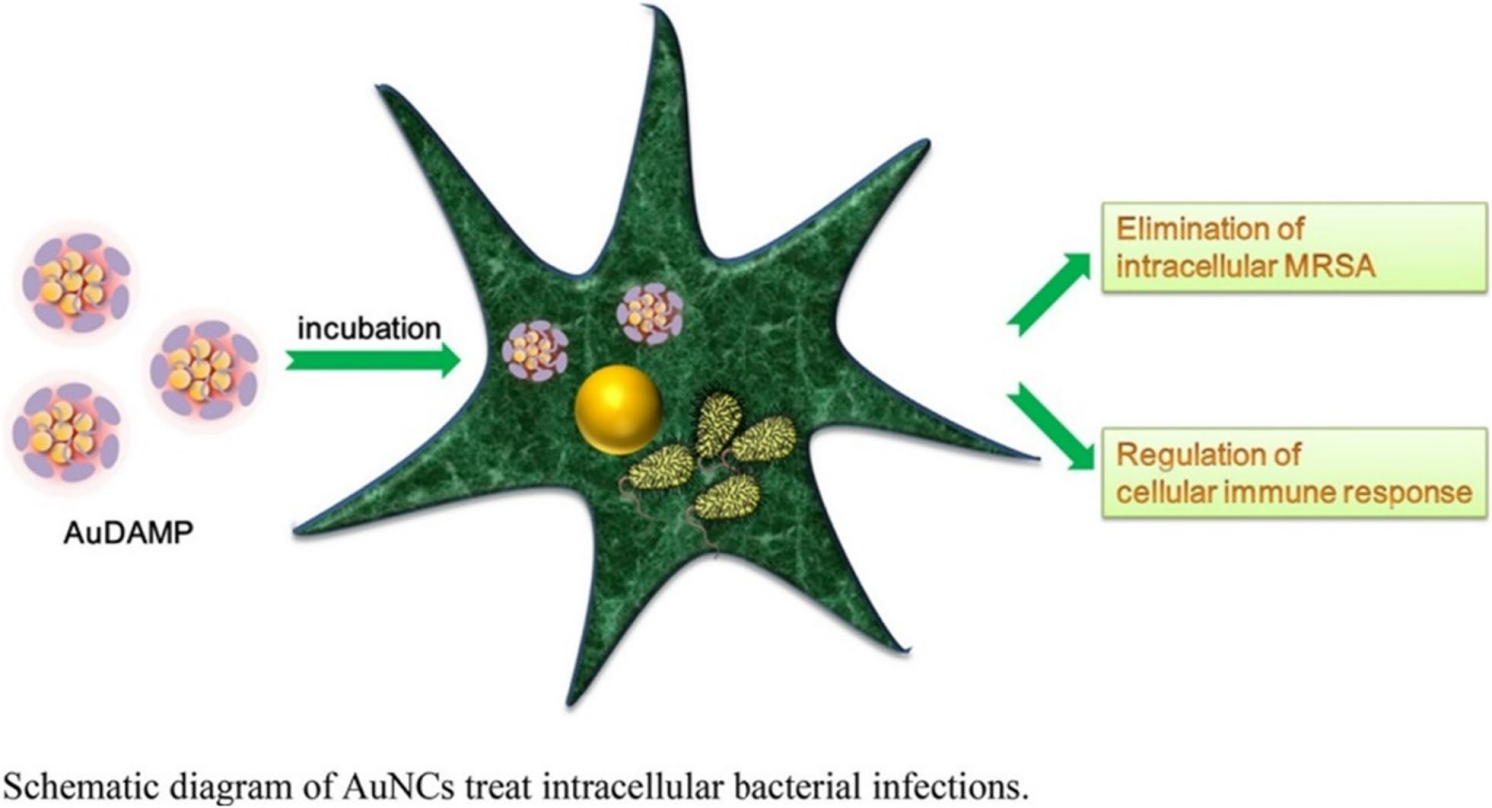
Schematic illustration of treatment of intracellular bacterial infections with gold nanoclusters conjugated with 4,6-diamino-2-mercaptopyrimidine hydrate (AuDAMP). Reproduced with permission from ref [115]. Copyright 2021 Elsevier

In addition to influencing ROS indicator genes, a recent report by Tang et al. provided new insights into ligand-conjugated metal NCs’ ability to control inflammatory receptors on macrophage cells as shown in Fig. 15. In order to observe the inflammatory response of macrophages during treatment of intracellular bacterial infections through essential inflammatory factors including NLR family pyrin domain-containing 3 (NLRP3), caspase-1, and interleukin (IL)-1, AuNCs conjugated with 4,6-diamino-2-mercaptopyrimidine hydrate (AuDAMP) were administered as treatment in that study. Findings demonstrated that AuDAMP prevented the occurrence of pyroptosis brought on by a bacterial infection while not causing an inflammatory response in macrophages themselves by inhibiting the inflammatory response of macrophages induced by an MRSA infection in a concentration-dependent manner. Additionally, AuDAMP treatment greatly raised the xenophagy level in macrophages, as seen by the increment of two protein markers of xenophagy, light chain 3 (LC3) and beclin-1. Accelerating the removal of intracellular bacterial infections and reducing the excessive inflammatory response of macrophages are both benefits of increasing autophagy [115]. In Tables 2 and 3, we respectively summarize examples of positive and negative surface ligands of metal NCs towards gram-positive and gram-negative bacteria.

**Table 2:** Effect of positively charged ligand-conjugated metal nanoclusters (NCs) towards gram-positive and gram-negative bacteria

Material	Target pathogens		Charge (zeta potential)	Results	Antibacterial mechanism	References
	Gram-positive bacteria	Gram-negative bacteria				
Gold nanoclusters (AuNCs)–DAMP	*Staphylococcus aureus* (ATCC29213) Methicillin-resistant *S. aureus* (MRSA) Vancomycin-resistant *Enterococcus* (VRE)	*Escherichia coli (*ATCC35218) Multidrug resistant (MDR) *E. coli* MDR *Acetobacter baumannii* MDR *Pseudomonas aeruginosa* MDR *Klebsiella pneumoniae*	+ 37.6 ± 1.1 mV	The antibacterial effect of AuNCs-DAMP widely ranges in gram-positive and gram-negative bacteria	Cell membrane damage	[93, 115]
AuNCs-MUTAB	*S. pneumoniae* *Bacillus subtilis* *Ent. Faecalis* VRE	*E. coli* *P. aeruginosa*	Positive (not mentioned)	The antimicrobial effect of AuNCs-MUTAB showed broad-spectrum actions against both in gram-positive and gram-negative bacteria	Damage to the membrane integrity of bacteria	[116]
Branched polyethyleneimine-functionalized silver nanoclusters (bPEI-AgNCs)	*Amycolatopsis azurea* MRSA (CD-489) *E. faecalis* (CD-746, CD-895) *S. aureus* (CD-1578)	*P. aeruginosa* (ATCC 19660, CD-1006, CD-23, CD-14) *E. coli* (CD-549, CD-2, CD-3) *Ent. cloacae* complex (CD-1412, CD-866)	+ 30 mV	bPEI-AgNCs selectively suppressed the growth of both gram-positive and gram-negative bacteria	Cell membrane disruption	[117]
Prot/MTU-AuNCs	*S. aureus*	*E. coli*	Positive (not mentioned)	Prot/MTU-AuNCs showed an antimicrobial effect against both gram-positive and gram-negative bacteria	Cell membrane damage and reactive oxygen species (ROS) generation	[101]
Peptide@AgNCs (KLA@AgNCs)	*S. aureus*	*E. coli*	+ 29.8 mV	The minimum inhibitory concentration (MIC) of KLA@AgNCs was determined in both gram-positive and gram-negative bacteria	Membrane integrity damage	[118]
AuNCs decorated with amine-functionalized graphene oxide (Au-GO-NH_2_) nanosheets	*S. aureus* *B. subtilis*	*E. coli* *P. aeruginosa*	+ 10.4 ± 0.5 mV	Cell viability and MIC level of Au-GO-NH_2_ were better for gram-positive bacteria than gram-negative bacteria. But the growth curve for gram-negative bacteria was lower than for gram-positive bacteria	Bacteria were captured in a film, oxidative stress was produced, and photothermal ablation occurred	[119]
Peptide-reduced gold nanoclusters (Au-HHC NCs)	*S. aureus* *S. epidermidis*	*E. coli* *P. aeruginosa*	+ 31.4 ± 5.7 mV	Au-HHC NCs (positive charge) exhibited higher antimicrobial activity than Au-HHC-CA NCs (negative charge). Au-HHC NCs showed low MICs toward gram-positive and gram-negative bacteria	Cell membrane disruption	[120]
Positively charged ligand-conjugated metal NCs which showed better antibacterial effects against gram-positive bacteria than against gram-negative bacteria						
Quaternary ammonia (QA) salt-functionalized AuNCs (QA-AuNCs)	*S. aureus* MRSA	*E. coli* MDR* E. coli*	Positive (not mentioned)	QA-AuNCs had a striking antibacterial effect on gram-positive and gram-negative bacteria but were better for gram-positive bacteria	Membrane integrity, membrane permeability, and membrane potential damage	[113]
Alkyl-thiolated zwitterionic and pyridinium ligands AuNCs	*S. aureus* MRSA	*E. coli* MDR *P. aeruginosa* MDR *K. pneumoniae*	Positive (not mentioned)	MIC levels of AuNCs were smaller for gram-positive bacteria than for gram-negative bacteria, at 8 µg/ml for *S. aureus* and MRSA, and 32 µg/ml for *E. coli, P. aeruginosa,* and* K. pneumoniae*	Adhesion and penetration of GNCs into the cell envelope	[109]
Riboflavin-protected silver nanoclusters (RF@AgNCs)	*S. aureus*	*E. coli*	+ 0.283 mV	RF@AgNCs showed an antimicrobial effect on both gram-positive and gram-negative bacteria, but were better for gram-positive bacteria. Relative viabilities of S*. aureus* and *E. coli* treated with RF@AgNCs were 0.83% and 2.08%	Cell membrane damage	[121]
AgNCs-GSH@chitosan	*S. aureus* *B. subtilis*	*E. coli* *P. aeruginosa*	+ 24.2 ± 4.7 mV	MIC levels of AgNCs-GSH@chitosan were smaller for gram-positive bacteria than for gram-negative bacteria, at 0.48 and 0.63 µM for *S. aureus* and *B. subtilis*, and 0.73 and 1.13 µM for *E. coli* and *P. aeruginosa*	ROS generation	[122]
Positively charged ligand-conjugated metal NCs which showed better antibacterial effect against gram-negative bacteria than gram-positive bacteria						
Dpep-AgNCs	*S. aureus*	*E. coli* *Shewanella oneidensis* MR-1	Positive (not mentioned)	Dpep-AgNCs had an antibacterial effect against both gram-positive and gram-negative bacteria but were better against gram-negative bacteria. MIC levels of Dpep-AgNCs for *E. coli* and *She. oneidensis* were 6.5 µM, but for *S. aureus* was 13 µM	Uptake and internalization of Ag ions; ROS generation	[80]
4,6-diamino-2-pyrimidinethiol (DAPT)-modified AuNCs (DAPT-AuNCs)	*S. aureus*	*E. coli*	+ 14 mV	Via SEM imaging, DAPT-AuNCs more strongly damaged gram-negative bacteria than gram-positive bacteria, but the ROS generation level of gram-positive bacteria was higher than that of gram-negative bacteria	Cell membrane damage	[123]

Dpep,; GSH,; CA, cinnamaldehyde; HHC,; KLA,; MTU,; Prot.,; MUTAB,; DAMP, 4,6-diamino-2-mersaptopyrimidine

**Table 3:** Effects of negatively charge ligand-conjugated metal nanoclusters (NCs) towards gram-positive and gram-negative bacteria

Material	Target pathogens		Charge (zeta potential)	Results	Antibacterial mechanism	References
	Gram-positive bacteria	Gram-negative bacteria				
Gold NCs (AuNCs)-MBA	*Staphylococcus aureus*	*Escherichia coli*	− 32.2 ± 3.4 mV	Au_25_NCs-MBA killed both gram-positive and gram-negative bacteria	Reactive oxygen species (ROS) formation increased when there was a metabolic imbalance that led to the overproduction of pro-oxidative enzymes and suppression of antioxidative enzymes	[61]
Au_x_Ag_25-x_(MHA)_18_ NCs	*S. aureus*	*E. coli*	− 36 to − 32 mV	The variation of nanoclusters showed a U-shaped antimicrobial trend for both gram-positive and gram-negative bacteria; Au-rich NCs had a decreased antimicrobial ability, while Ag-rich NCs had an increased antimicrobial ability	ROS generation, inducement of oxidative stress, regular metabolism interference	[124]
Au_25_MHA_18_NCs Au_25_Cys_18_NCs Au_25_Cystm_x_MHA_18-x_NCs Au_25_MetH_x_MHA_18-x_NCs Au_25_MBA_18_NCs	*S. aureus*	*E. coli*	− 20.5 ± 4.1 to − 37.4 ± 2.5 mV	By adjusting both the type and ratio of surface ligands on AuNCs, negatively charged AuNCs produced more ROS, resulting in greater gram-positive and gram-negative bacterial killing efficiencies	Cell uptake, NC internalization, ROS generation	[111]
(Au_25_Cystm_1-4_MHA_17-14_) on Holmium ions (Ho)-graphene oxide (GO) nanosheets	*S. aureus*	*E. coli*	− 37.6 mV	Ho-GO-AuNCs killed both gram-positive and gram-negative bacteria	Cell uptake, NC internalization, ROS generation	[125]
Ag-GSH-NCs encapsulated with liposomes	*S. aureus* *B. subtilis*	*E. coli* *Pseudomonas aeruginosa*	− 29.3 ± 0.8 mV	Ag-GSH-NCs killed both gram-positive and gram-negative bacteria	ROS generation	[126]
AuNCs-MHA	*Cloistridioides difficile*	–	Negative (not mentioned)	AuNCs-MHA killed *C. difficile* without causing a significant toxic effect on human cells	Metabolic imbalance, ROS generation	[127]
AuNCs-MHA	–	*Shigella* (Sf301, R2448 & RII-1)	Negative (not mentioned)	AuNCs-MHA represented a good potential alternative to antibiotics to treat *Shigella* infections	Cell membrane damage, ROS generation	[128]
GSH-AuNCs	–	*Acetobacter aceti*	Negative (not mentioned)	Antibacterial activity increased with the concentration of GSH-AuNCs, as demonstrated by bacterial growth curves	ROS generation	[129]
Negatively charged ligand-conjugated metal NCs which showed better antibacterial effects against gram-negative bacteria than gram-positive bacteria						
AuNCs-MHA	*S. aureus* *B. subtilis*	*E. coli* *A. baumannii*	Negative (not mentioned)	The antibacterial effect of AuNCs-MHA worked better against gram-negative bacteria than gram-positive ones. MIC levels for *E. coli* and *A. baumannii* were 50 and 200 µM while for *S. aureus* and *B. subtilis*, both required > 200 µM	Interactions with the phospholipid bilayer, cytosolic protein binding, ROS generation	[76]
Luminescent copper NCs (CuNCs)-doped hydroxyapatite nanoparticles (HAP NPs)	*S. aureus* (MTCC 96) *B. subtilis* (MTCC 1305)	*E. coli* (DH5α) *P. aeruginosa* (MTCC 2488)	− 3.14 ± 0.17 mV	It was discovered that the kanamycin-loaded doped HAP NPs were more efficient against gram-negative bacteria than gram-positive bacteria	Formation of hydroxyl radicals, depletion of NADH, cell damage	[100]
Dihydrolipoic acid-stabilized dual-functional silver NCs (DHLA-AgNCs)	*S. aureus*	*E. coli*	− 28.8 mV	Results showed that DHLA-AgNCs exhibited excellent antibacterial activities against gram-negative but had no apparent antibacterial activity against gram-positive bacteria	Cell membrane damage	[130]
Negatively charged ligand-conjugated metal NCs which showed better antibacterial effects against gram-positive bacteria than gram-negative bacteria						
AuNCs-MBA	*S. aureus*	*E. coli*	− 36 + 2.3 mV	AuNCs-MBA generated higher ROS levels for gram-positive bacteria than gram-negative bacteria	Physical absorption into the cell membrane, metabolic imbalance, ROS generation	[131]
Au_25_(MBA)_18_ NCs on MXene nanosheets	*S. aureus*	*E. coli*	− 16.8 mV	Both gram-positive and gram-negative bacteria were eventually killed due to synergistic physical (through MXene) and chemical (via MXene and AuNCs) antibacterial processes; however, the outcome was marginally more beneficial for gram-positive bacteria	Cell membrane damage, ROS generation	[132]
Thiol-terminated phosphorylcholine (PC-SH)-protected silver NCs (PC-AgNCs)	*S. aureus*	*E. coli*	Negative (not mentioned)	PC-AgNCs worked against both gram-positive and -negative bacteria, but were better against gram-positive bacteria	Cell membrane damage, ROS generation	[133]
Photobactericidal polymer containing crystal violet (CV) and thiolated gold NCs (Au_25_(Cys)_18_)	*S. aureus*	*E. coli*	− 31.8 mV	By white light, the materials worked in both gram-positive and gram-negative bacteria but were better for gram-positive bacteria	Promotion of hydrogen peroxide (H_2_O_2_) and ROS generation	[134]

MBA, 3-mercaptobenzoic acid; MHA, 3-mercaptohexanoic acid; Cys, cysteine; Cystm, cysteamine hydrochloride; MetH, 2-mercaptoethanol; GSH, glutathione; NADH, nicotinamide adenine dinucleotide; MXene,

## Challenges and future perspectives

As an innovative type of versatile nanomedicine, metal NCs have been recently found to possess great potential as antibacterial agents, particularly against antibiotic-resistant bacteria. This study demonstrated that the unique physicochemical properties of metal NCs, such as a large surface area, ultrasmall size, and easy surface modification, make them attractive candidates for combating bacterial infections. Researchers are exploring new synthesis methods and design strategies to achieve better control over these fields, leading to the development of more advanced and tailored materials [50, 135, 136]. For examples, the integration of metal NCs with biomolecules such as DNA, small molecules, peptides, dendrimers, and proteins will offer opportunities for the development of hybrid systems with enhanced functionalities. In addition, the investigation of metal NCs due to their unique optical and electronic properties will enhance performance in bio-applications, such as bioimaging, sensing, and drug delivery. Due the physicochemical properties and application efficacy of metal NCs are extremely sensitive to their components, it provides an excellent opportunity to comprehend the role in determining their antibacterial activity, as well as the composition-related properties and applications, which necessitates multidisciplinary collaborative research.

Regarding the size of the metal NCs, the smaller the size of the metal NCs, the greater the ability to penetrate bacterial cell bodies. The smaller the size of the material will result in a higher ratio of surface area to volume than larger sizes, making them more toxic. With their small size, metal NCs can be easily internalized by bacteria via simple diffusion through small pores in the cell wall. Metal NCs also can imitate natural enzymes such as peroxidase-like or oxidase-like activity that converts hydrogen peroxide into ROS capable of killing both gram-positive and gram-negative bacteria. The smaller the particle size will increase the number of metal atoms on the surface leading to a rise in the catalytic activity of the particles. The size of the metal NCs also can directly impact interactions of NCs with their nano-bio interface. Due to their higher diffusivity, metal NCS could enter the interior of the bilayer more quickly than their large counterparts. Surface ligands engineering can help to create high specifity and enhance nano-bio interaction between the metal NCs and the bacterial cell wall. Cationic surface, on the other hand, shows greater bacterial killing effectiveness because they can initiate interactions with bacterial cells that allow them to insert their hydrophobic tail into the bacterial cell wall. The strong positive charge on the surface of metal NCs allows the promotion of electrostatic adsorption onto bacterial surfaces, resulting in the effective internalization of metal NCs into bacteria and the accumulation of ROS that cause bactericidal action.

However, metal NCs as antibacterial agents have various obstacles in meeting biocompatibility requirements for medical applications [137], such as resolving the stability and ADMET (absorption, distribution, metabolism, excretion, and toxicity) properties of complexes metal NCs [40, 138], understanding the dynamic nano-bio interaction as the effect of surface ligand chirality and isomerization on metal NCs on biological interactions of nanomaterials [23, 139], as well as a high sensitivity or selectivity to lesion areas or tumor-targeting, which has implications for diagnostic and therapeutic efficacy. Although some metals, such as Au, are bio-inert, doping toxic species (such as Hg and Cd) in the metal core of NCs may cause significant cytotoxicity. However, the cytotoxicity of metal NCs can be minimized by engineering their ligands with hydrophilic anti-oxidative surface ligands, grafting biocompatible compounds, and modulating ligand chirality. Nonetheless, the Community for Open Antimicrobial Drug Discovery (CO-ADD) evaluated a screened the large number of metal complexes in 2020, comparing their capacity to limit bacterial growth to that of purely organic molecules. Approximately 295,000 compounds, including almost 1,000 metal-containing ones, CO-ADD analysis found that metal-containing compounds had hit rates against important ESKAPE pathogens that were more than ten times greater than the remainder of the molecules in the CO-ADD database. Importantly, there was no significant difference in the frequency of cytotoxicity for mammalian cell lines and haemolysis for human red blood cells between the two classes of substance for either toxicity test [140]. One example of a metal nanocluster, AuNCs, apparently does not produce cytotoxicity in mammalian cells, and the ROS production within it simply leads to increased cellular metabolism and proliferation rather than DNA damage and cell death [141]. Another example shown that AuNCs with DAMP ligands have antibacterial potential but are also safe and can even promote macrophage autophagy, speed the clearance of intracellular bacterial infections, and reduce macrophage inflammatory response [115]. The technique for managing metal NCs biocompatibility can also be expanded by employing the alloying process to improve both the structural stability and luminescence intensity of metal NCs. Therefore, the future research aims to improve their biocompatibility, stability, and targeting capabilities for more effective and safe biomedical applications [52].

In addition, another challenging factor to address is the effectiveness of metal NCs in combating the antibiotic resistance. Metal complexes having antibacterial characteristics are the most likely candidates to progress toward clinical approval. Metal ions are required by all living organisms. Metal-ion-containing enzymes catalyze about 50% of all metabolic reactions in bacteria, requiring the cell to maintain homeostasis for critical metals at sufficiently high levels to meet cellular demands while remaining poisonous [138]. Metal-based antibiotics are great prospects for outsmarting bacterial defense and resistance mechanisms due to their vast structural diversity and alternate or multimodal modes of action. Metal-based antibiotics can develop narrow-spectrum medicines that target certain bacterial infections by using the routes of metallophore uptake specificity or linker cleavage specificity. This eliminates one of broad-spectrum antibiotics drawbacks: their capacity to promote the emergence of resistance by selecting for resistant mutants in non-pathogenic commensal bacteria, which then transfer this resistance to pathogenic strains [138]. Metal ions from metal NCs can be tuned to take the same pathways within biological processes using the same idea. Furthermore, it has been reported that the combination of metal NCs used as nanocarriers for the delivery of existing antibacterial agents can broaden the antibacterial spectrum of conventional antibiotics and achieve controlled or targeted drug release, thereby augmenting the therapeutic effect and reducing side effects [23].

The creation of biofilm, which acts as a natural barrier to drug penetration and activation, is also an important obstacle that must be resolved in the treatment of antibiotic resistance. Surface modification of metal NCs can also be used to combat bacterial biofilms [23]. For example, the surface-assembled nanoantibiotics (rAgNAs), a customized pH-sensitive charge reversal ligands, were used as developed a pH-responsive biofilm elimination strategy. This metal NCs group has been shown to selectively trigger antibacterial activity in the acidic biofilm milieu [142]. Furthermore, cationic thiolate modified AuNCs have a highly effective antibacterial action against mature biofilm, which is most likely due to the high penetration of positively charged AuNCs into biofilm [116]. DNase-functionalized AuNCs can hydrolyze DNA in the extracellular polymeric material matrix and generate oxidative stress via photoactivation to eliminate biofilm [143].

However, treating these antibiotic resistance situations requires a concerted effort by the community to solve the issues stated above, because otherwise, this case would be difficult to proceed. To address this global health challenge, complex issues concerning antibiotics, such as their overuse and misuse by patients and doctors, their widespread use in agriculture, and their low return on investment for drug companies (several biotech companies that have succeeded in bringing an antibiotic to market have gone bankrupt afterwards due to poor sales), must be addressed. Careful stewardship is also required to ensure that drug-resistant infections remain effective for as long as possible [138]. Overall, a significant amount of effort need to be done to increase the antibacterial activity of metal NCs in order to meet the requirements in clinic application. With the continuous efforts of the research community, we can endow metal NCs with new physical, chemical, and biological properties that can be used to construct antibacterial agents for various applications, benefiting translation nanomedicine and ushering in a new era of atomic-level precision nanomedicine.

## Data Availability

Not applicable.
